# Results of the ECHO (Eating habits CHanges in Oncologic patients) Survey: An Italian Cross-Sectional Multicentric Study to Explore Dietary Changes and Dietary Supplement Use, in Breast Cancer Survivors

**DOI:** 10.3389/fonc.2021.705927

**Published:** 2021-11-01

**Authors:** Greta Caprara, Maria Tieri, Alessandra Fabi, Valentina Guarneri, Cristina Falci, Maria Vittoria Dieci, Monica Turazza, Bettina Ballardini, Alessandra Bin, Saverio Cinieri, Patrizia Vici, Emilia Montagna, Claudio Zamagni, Cristina Mazzi, Alessandra Modena, Fabiana Marchetti, Matteo Verzè, Francesca Ghelfi, Lucilla Titta, Fabrizio Nicolis, Stefania Gori

**Affiliations:** ^1^ Department of Experimental Oncology, Istituto Europeo di Oncologia (IEO), European Institute of Oncology, Istituto di Ricovero e Cura a Carattere Scientifico (IRCCS), Milano, Italy; ^2^ Fondazione Tera, Novara, Italy; ^3^ Medical Oncology 1 - Istituto Nazionale Tumori Regina Elena Istituto di Ricovero e Cura a Carattere Scientifico (IRCCS), Roma, Italy; ^4^ Precision Medicine in Breast Cancer Unit, Fondazione Policlinico Universitario A. Gemelli Istituto di Ricovero e Cura a Carattere Scientifico (IRCCS), Roma, Italy; ^5^ Department of Surgery, Oncology and Gastroenterology, University of Padova, Padova, Italy; ^6^ Medical Oncology 2 - Istituto Oncologico Veneto Istituto Oncologico Veneto (IOV) Istituto di Ricovero e Cura a Carattere Scientifico (IRCCS), Padova, Italy; ^7^ Department of Oncology, Istituto di Ricovero e Cura a Carattere Scientifico (IRCCS) Sacro Cuore Don Calabria Hospital, Negrar di Valpolicella, Italy; ^8^ Breast Division, MultiMedica Breast Unit Multimedica Istituto di Ricovero e Cura a Carattere Scientifico (IRCCS), Milano, Italy; ^9^ Dipartimento di Oncologia, Azienda Sanitaria Universitaria Integrata di Udine, Udine, Italy; ^10^ Unità Operativa Complessa di Oncologia Medica, ASL Brindisi Senatore Antonio Perrino Hospital, Brindisi, Italy; ^11^ Unità Operativa Semplice Dipartimentale (UOSD), Sperimentazioni di FASE IV, Istituto Nazionale Tumori Regina Elena Istituto di Ricovero e Cura a Carattere Scientifico (IRCCS), Roma, Italy; ^12^ Division of Medical Senology, Istituto Europeo di Oncologia (IEO), European Institute of Oncology, Istituto di Ricovero e Cura a Carattere Scientifico (IRCCS), Milano, Italy; ^13^ Medical Oncology Unit, Istituto di Ricovero e Cura a Carattere Scientifico (IRCCS) Azienda Ospedaliero-universitaria di Bologna, Bologna, Italy; ^14^ Clinical Research Unit, Istituto di Ricovero e Cura a Carattere Scientifico (IRCCS) Sacro Cuore Don Calabria Hospital, Negrar di Valpolicella, Italy; ^15^ Medical Direction, Istituto di Ricovero e Cura a Carattere Scientifico (IRCCS) Sacro Cuore Don Calabria Hospital, Negrar di Valpolicella, Italy; ^16^ Fondazione De Marchi-Department of Pediatrics, Istituto di Ricovero e Cura a Carattere Scientifico (IRCCS) Ca’ Granda Ospedale Maggiore Policlinico, Milano, Italy; ^17^ The Need For Nutrition Education/Innovation Programme (NNEdPro) Global Centre for Nutrition and Health, St John’s Innovation Centre, Cambridge, United Kingdom; ^18^ Associazione Italiana di Oncologia Medica (AIOM) Foundation Past President, Medical Direction, Istituto di Ricovero e Cura a Carattere Scientifico (IRCCS) Sacro Cuore Don Calabria Hospital, Negrar di Valpolicella, Italy; ^19^ Associazione Italiana di Oncologia Medica (AIOM) Foundation President, Department of Oncology, Istituto di Ricovero e Cura a Carattere Scientifico (IRCCS) Sacro Cuore Don Calabria Hospital, Negrar di Valpolicella, Italy

**Keywords:** eating habits survey, health information, dietary changes, cancer patients, cancer survivors, breast cancer

## Abstract

The role of a healthy diet in cancer prevention is well recognized. Recent data indicate that following the same advices can also improve cancer survivors’ quality of life. Breast cancer (BC) patients are commonly concerned about diet and nutrition and frequently express the need to obtain health-related information and the will to change their diet and lifestyle. Hence, be aware of survivors’ dietary changes and information needs is crucial for healthcare professionals to guide them toward optimal lifestyle choices. In order to investigate eating habits changes in a BC survivors’ population, we conceived the cross-sectional multicentric study ECHO (Eating habits CHanges in Oncologic patients) Survey. Data were collected from 684 patients, diagnosed with invasive breast cancer, in order to investigate their changes in food consumption, use of supplements, or the beginning of a specific diet, after BC diagnosis. We also examined the sources of information used and if any modification in their diets was reported to the oncologist. We primarily observed that patients increased their consumption of vegetables, pulses, nuts, fruits, wholemeal bread/pasta, grains and fish; while decreasing red and processed meat, refined bread/pasta, baked good and animal fat consumption. Survivors also reported the use of dietary supplements, mainly vitamins, aimed at counteracting therapies’ side effects. Changes in nutritional habits were often adopted without asking or informing the oncologist. Despite BC survivors made some positive changes in their nutritional habits, those modifications were mostly pursued by less than half of them, while the majority of patients consumed nutritional supplements after diagnosis. These results, as well as the failure to communicate with the physicians, reinforce the need to both improve the patient-healthcare professional relationship and to develop tailored nutrition counselling and intervention programs for cancer survivors.

## Introduction

The term “cancer survivor” refers to a person who has been diagnosed with cancer, regardless of the course of the disease (before, during or after treatment). An individual is considered a cancer survivor from the time of diagnosis, through the rest of his/her life (1, 2).

In 2020, the global cancer burden has risen to around 19.3 million cases worldwide and this number is projected to increase to about 30.2 million, in 2040 (3). The growth and aging of the population, along with advances in early detection and treatment, will led to a continuous increase in cancer survivors, making them a significant part of the current and future population. According to Globocan 2020, more than 50 million people worldwide are living within 5 years of a past cancer diagnosis (5-year prevalence): breast cancer (BC) patients represent a relevant part of this survivors group, with an incidence and a 5-year prevalence rate of 11.7% and 15.4%, respectively (3).

In 2020, 3.6 million people, corresponding to 6% of the total population, were estimated to live in Italy after a cancer diagnosis, a number which has risen by 36% in the past 10 years (4). Breast cancer is the most frequent cancer, accounting for 14.6% of all new cancer diagnoses and recording the highest incidence among Italian women (30.3%) (4).

Nowadays, the role of diet and lifestyle in cancer prevention is well established, indicating tobacco smoking, overweight, obesity, unhealthy diets, alcohol consumption and insufficient physical activity among the major risk factors for tumor development (5, 6). Indeed, mounting evidence suggests that more than 50% of cancers can be prevented simply applying evidence-based prevention strategies, such as healthy lifestyle behaviors, screening programs and vaccinations (7). Notably, overweight and obesity are casually linked to the development of 12 different kind of cancers, including post-menopausal BC. Several studies have shown positive associations between adult body mass index (BMI), waist circumference and body-weight gain, in adulthood and postmenopausal breast cancer, especially for estrogen-receptor-positive tumors (8, 9). Furthermore, accumulating evidence suggests that elevated body fatness is a predictor of poor outcome in BC survivors: chronic obesity-associated inflammation may, in fact, impair the efficacy of treatments (10), enhance disease progression (11, 12) and increase the risk of other chronic pathologies, which in turn can contribute to reduce patients’ overall survival. Therefore, despite evidence is still inadequate to make specific recommendations, Continuous Update Project (CUP) Expert Panel of the World Cancer Research Fund (WCRF) International and the American Institute for Cancer Research (AICR), reckoned that, unless otherwise advised by a health professional, BC survivors are strongly encouraged to reach/maintain a healthy weight (13–15).

Beyond the impact of body fatness, the combination of specific dietary components, as well as physical activity, are able to affect the susceptibility to cancer development. This effect has been demonstrated by the European Prospective Investigation into Cancer and Nutrition (EPIC) study (16), one of the largest cohort studies in the world, and by the CUP of the WCRF/AICR, which conducted the most rigorous analyses of the published literature linking cancer risk to diet, nutrition and physical activity (5). Briefly, they showed that an overall dietary pattern based on the regular consumption of plant-based foods (vegetables, fruits, legumes, wholegrains, etc.), a moderate amount of fish, dairy and poultry and a low consumption of red and processed meat, salt preserved foods, sugars, alcohol and pastry can decrease the risk of tumor development (5). Despite data are still inadequate to make specific recommendations, many dedicated organizations have established that following the same general nutritional advice could be appropriate even for cancer survivors (5, 14, 15, 17, 18). More interesting, some recent works indicate that healthy lifestyle and proper diet are also associated with better health-related quality of life in cancer survivors (19, 20). However, further studies are required to both understand the relationship between specific lifestyle factors and favorable prognosis and to design behavioral guidelines for those patients (20).

Mounting insight into cancer metabolism and treatment is also underlining the importance of diet and specific nutrients in both limiting drug-induced side effects and tumor therapeutic response (21, 22). Nonetheless, considering the high heterogeneity of the different kind of cancers, there is still much to be learned before being able to develop evidence-based tailored nutritional interventions for all the existing tumor treatment options (23, 24).

Due to both the worldwide frequency of BC, which is currently the leading cause of global cancer incidence (25), and the increase in the long-term survivors, the impact of diet and lifestyle behaviors on BC risk, recurrence and survival rates has been widely investigated. CUP Expert Panel of WCRF/AICR showed strong evidence linking (i) alcohol consumption and (ii) body fatness - for postmenopausal women - with an increased risk of developing BC. On the other hand, they described strong evidence connecting physical activity and limited but suggestive evidence correlating (i) non-starchy vegetable - for oestrogen receptor negative breast cancer -, (ii) food containing carotenoids, (iii) diets high in calcium and (iv) dairy products - for premenopausal women - with a lower risk of BC incidence (26).

Even though the emerging evidence is still not strong enough to make specific recommendations, in women diagnosed with BC, WCRF/AICR depicted limited but suggestive evidence linking (i) a healthy body weight, (ii) being physically active, (iii) eating foods containing fiber, (iv) eating foods containing soy and (v) a lower intake of total fat (in particular saturated fat), with a better prognosis (15). Accordingly, the American Cancer Society (ACS)/American Society of Clinical Oncology (ASCO) Breast Cancer Survivorship Care Guideline recommends that primary care clinicians advice survivors to (i) engage in regular physical activity, avoid inactivity and return to normal daily activities as soon as possible after diagnosis; (ii) achieve a dietary pattern that is high in vegetables, fruits, whole grains, and legumes, low in saturated fats and limited in alcohol consumption; (iii) achieve and maintain a healthy weight; (iv) limit the consumption of high‐calorie foods and beverages and increase physical activity to promote and maintain weight loss, if overweight or obese (27).

Frequently, cancer diagnosis is associated to the so called “teachable moment”, an appropriate condition when survivors may be more receptive to adopt beneficial lifestyle changes and improve their health (28). In agreement with this, we have previously described that many cancer survivors are willing to change their eating habits: in particular, from 30% to 60% of BC patients are highly motivated to modify their diet (29). Most of the reported changes referred to an increase in fruit and vegetable consumption and a reduction in red meat, fat and sugary food intake. Younger age, higher education, and longer time from diagnosis were more likely associated with the described dietary modifications. Changes were primarily made in order to relieve side effects of cancer therapies, cure the disease or avoid cancer recurrence. Moreover, many survivors have been reported to frequently use a variety of dietary supplements (29). In summary, diet and nutrition tasks are recognized as common concern of cancer survivors, which frequently express both the need to obtain health-related information and the will to change their diet and lifestyle to prevent tumor recurrence (30, 31). Therefore, healthcare professionals need to be informed about both patients behavioral changes and where they collect information, in order to guide them toward optimal lifestyle choices, in line with proper recommendations (32, 33).

To investigate eating habits changes in Italian breast cancer survivors, we have conceived a cross-sectional multicentric study, the ECHO (*Eating habits CHanges in Oncologic patients*) Survey. An *ad-hoc* questionnaire was developed and validated with a pre-test (29, 34). We first aimed to explore and report dietary changes and supplement use in patients with invasive breast cancer (stages I-II-III). Then we identified and discussed the sources of information mostly accessed by BC survivors, which may have influenced the changes referred. Finally, we inspected whether the modifications in their dietary habits have been reported to the oncologist.

## Material and Methods

### Study Design and Patients

The ECHO (Eating habits CHanges in Oncologic patients) study was coordinated by the Medical Oncology center IRCCS Sacro Cuore Don Calabria Hospital, Negrar di Valpolicella, Verona, Italy. Other centers participated to the study (Istituto Oncologico Veneto, Padova, Italy; Senatore Antonio Perrino Hospital, Brindisi, Italy; INT - Regina Elena, Medical Oncology 1, Roma, Italy; INT - Regina Elena, Medical Oncology 2, Roma, Italy; Azienda Sanitaria Universitaria Integrata, Udine, Italy; Istituto Europeo di Oncologia, Milano, Italy; Breast Unit Multimedica, Milano, Italy; Azienda ospedaliero-universitaria Policlinico Sant’Orsola-Malpighi, Bologna, Italy) for a total of 9 medical oncology centers involved (**Supplementary Table 1**). The inclusion criteria were based on: (i) patients’ willingness to participate and general condition allowing understanding of the questions and purpose of the study; (ii) histopathological diagnosis confirming breast cancer; (iii) performance status 0-1; (iv) normal organ and bone marrow function and (v) no previous cancer diagnosis. The exclusion criteria include the following: (i) inability to answer questions; (ii) pregnancy or breast-feeding; (iii) clinically significant psychiatric, neurological, or medical disorders and (iv) history of alcohol or drug abuse.

Enrolled patients, who visited the 9 oncology centers for treatments or follow-up, were all adults (older than 18 years) and diagnosed with invasive breast cancer (stages I-II-III), 1-24 months prior to enrolment. All subjects signed an informed consent form. Participants were provided with all the information in writing and they reserved unconditional or absolute right of withdrawal at any time and without giving any reason. Detailed patient characteristics and the histopathological features of breast cancer subtypes are reported in **Tables 1** and **2**, respectively.

**Table 1:** Demographic and clinical characteristics of overall patient sample (N = 684).

Demographic and clinical characteristics	Patients (N = 684)	
	No.	(%)
Time since diagnosis, months		
1 - 6	294	(43.0)
7 - 12	168	(24.6)
13 - 18	108	(15.8)
19 - 24	114	(16.7)
Age, years		
<50	229	(33.5)
50 - 64	288	(42.1)
≥65	156	(22.8)
Missing	11	(1.6)
Sex		
Female	672	(98.3)
Male	3	(0.4)
Missing	9	(1.3)
Education		
Primary or middle school	204	(29.8)
High school	304	(44.4)
University degree or higher	135	(19.7)
Missing	41	(6.0)
Cancer therapies		
None	24	(3.5)
Surgery	349	(51.0)
Radiotherapy	290	(42.4)
Chemotherapy	409	(59.8)
Hormone therapy	312	(45.6)

N, number of total respondents.

**Table 2:** Histopathological features of breast cancer subtypes.

	Totals (N = 684)	
	No.	(%)
Tumor histotype		
Invasive ductal carcinoma (IDC)	543	(79.4)
Invasive lobular carcinoma (ILC)	56	(8.2)
Other	60	(8.8)
Missing	25	(3.6)
Grading		
G1	69	(10.1)
G2	264	(38.6)
G3	266	(38.9)
Not known	42	(6.1)
Missing	43	(6.3)
Hormone receptors		
Negative	148	(21.6)
Positive	503	(73.6)
Not known	3	(0.4)
Missing	30	(4.4)
HER2 status		
Negative	431	(63.0)
Positive	211	(30.9)
Not known	2	(0.3)
Missing	40	(5.8)
KI67, mean ± SD	30 ± 21	
KI67		
≤20	264	(38.6)
>20	295	(43.1)
Missing	125	(18.3)

N, number of total respondents.

### *Ad-Hoc* Questionnaire Design

A pre-tested (34, 35) *ad-hoc* paper questionnaire with 39 questions was submitted to 685 breast cancer patients, from 1 December 2017 to 30 June 2019. The ECHO questionnaire was completed anonymously and administered by trained personnel, which did not intervene in the survey completion. The questionnaire consisted of four sections: (i) personal data and therapies, (ii) supplements use and specific diets followed after diagnosis, (iii) dietary changes after diagnosis and (iv) beliefs about the relationship between diet and cancer. In the third section of the questionnaire, patients have been asked to report whether their consumption of each of the 24 food indicated items (grouped into 9 categories) had either remained the same, started, increased, decreased, stopped, or if a specific food has never been eaten. Moreover, the questionnaire investigated the sources of information used by the patients and whether any modifications in their dietary habits was reported to the oncologist (use of supplements, dietary changes or following a specific diet).

In order to perform a descriptive analysis of the hypothetical correlations existing among clinical and biopathological characteristics of the tumor, pharmacological treatments and dietary changes, the oncologist had to fill a form (the full English translated version of it is provided in **Supplementary File 1**), for each assigned questionnaire, reporting: (i) tumor biopathology and histology features, (ii) disease staging and (iii) administered and/or current cancer treatment. Moreover, health care professionals recorded body weight and height, in order to calculate patients’ BMI. Of the 685 collected questionnaires, only one was excluded from the study because no answers were provided. Therefore, the final sample was represented by 684 breast cancer patients. The full English translated version of the ECHO questionnaire is provided in the Supplementary material as **Supplementary File 2**.

### Statistical Analysis

Data were summarized using numbers and percentages. The χ2 test was employed to compare categorical data. Multivariable logistic regression was used to identify variables associated with changes in food consumption. The logistic regression outcomes were obtained converting the 6-point Likert scale, describing the changes in food consumption, into a dichotomous variable. The responses “started”, “increased”, “decreased” and “stopped” were recorded as “changes in food consumption”, while “remained the same” and “have never eaten” were recorded as “no changes in food consumption”. The foods considered as outcome for the models were: red and processed meat, white meat, fresh fruit, vegetables, pulses and nuts, milk and cheese, eggs, baked goods and refined bread and pasta, fish and shellfish, homemade cakes/desserts and soft drinks, wholemeal bread or pasta and grains, preserved fish, animal fats and alcoholic drinks. All regression analyses were performed on the complete cases defined by the selected factors of interest, i.e.: BMI, age, education, time since diagnosis, menopausal status, HER2 status after treatment, tumor histotype after treatment, types of breast cancer surgery, types of adjuvant therapy and postoperative radiotherapy. Univariable regression analysis was performed on the selected variables and only the ones significantly associated with a change in food consumption (p-value < 0.2) were included in the full logistic regression model. All multivariate models were adjusted for participating oncology center. Model-building strategies included checking for assumptions for logistic regression analysis, checking for collinearity and stepwise forward and backward selection, based on the Akaike information criterion (AIC). Statistical analyses were performed using R software, version 4.0.2 (36). A statistical significance level of 0.05 was adopted for all tests.

## Results

### Changes in Food Consumption, After BC Diagnosis

**Figure 1:**
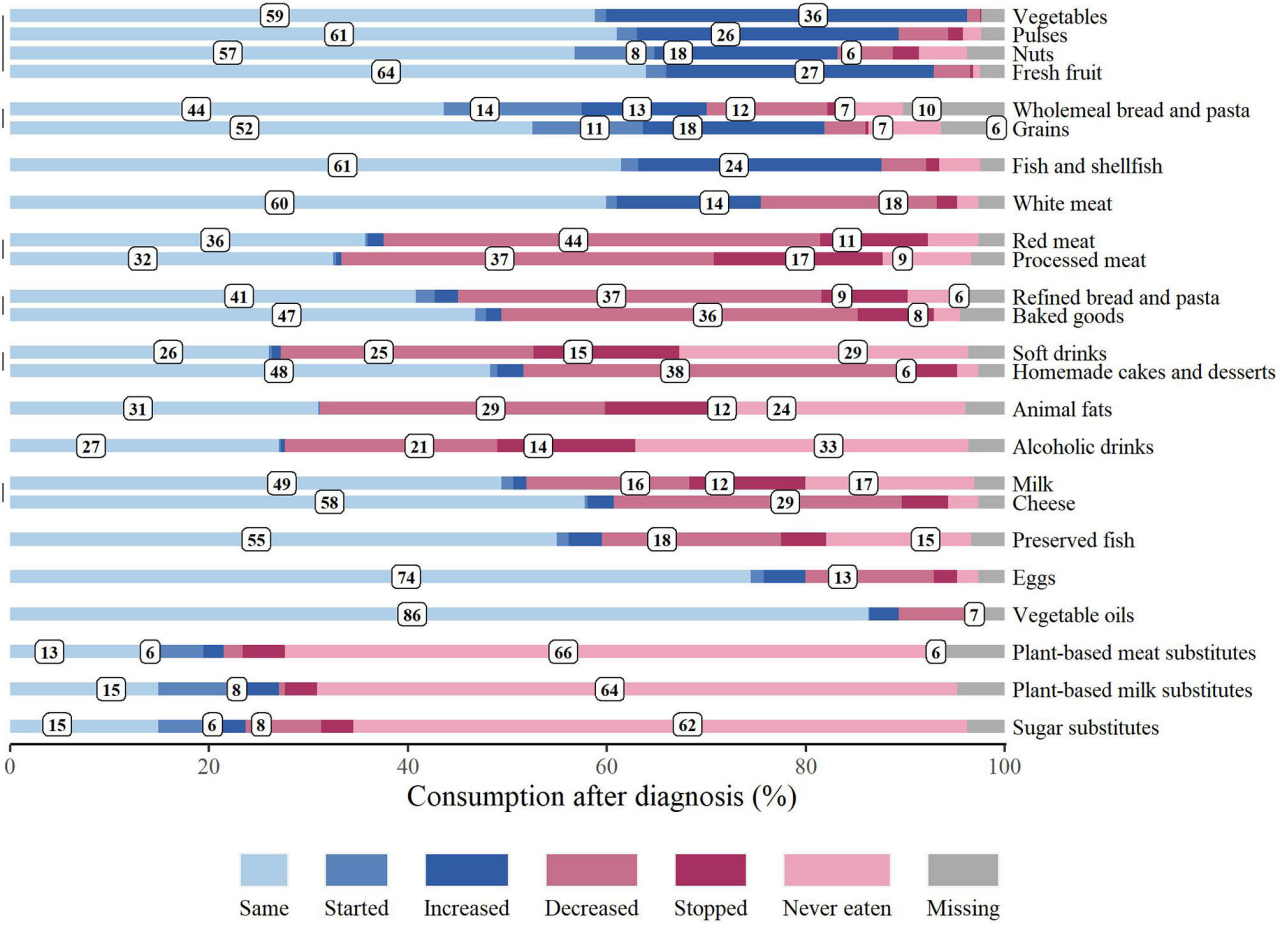
Changes in food consumption after breast cancer diagnosis. Food consumption patterns, reported by respondents, are expressed in percentage. “Missing” indicates that no responses were provided.

pt?>In order to investigate whether survivors reported any changes in dietary habits, we asked them to state if and how their consumption of 24 specific food items varied or not, after BC diagnosis. As illustrated in **Figure 1**, food consumption has been assessed as “same”, “started”, “increased”, “decreased”, “stopped” or “never eaten”. In agreement with the WCRF recommendations, the consumption of vegetables (36.3%), pulses (26.3%), nuts (18.4%) and fresh fruit (26.9%) increased, and the 8.0% of respondents started to eat nuts. Moreover, the 12.6% and the 18.3% of patients enhanced their consumption of wholemeal bread/pasta and grains, respectively, while the 13.9% and the 11.1% began to consume them. A 24.4% increase in fish and shellfish consumption was also reported, while 18.0% of the respondents decreased the intake of preserved fish. Still consistent with the guidelines for cancer prevention, the 43.9% and the 37.4% of survivors, decreased the consumption of red and processed meat, respectively. Notably the 10.8% and the 17.0% of respondents stopped eating those food items. On the other hand, 14.5% of patients increased white meat consumption, while the 17.7% decreased it. Refined bread/pasta and baked goods were also less consumed by the 36.6% and the 35.8% of the patients, respectively, and 37.9% of the respondents decreased homemade cakes and desserts’ consumption. In agreement with those data, the 28.7% and the 12.0% of survivors decreased and stopped animal fats’ consumption, respectively. Soft and alcoholic drink intake was decreased by the 25.4% and 21.3%, respectively, and the 14.6% and the 13.9% of the respondents stopped drinking them. Almost 30% of the patients decreased cheese consumption, the 16.4% diminished milk intake and the 11.7% stopped drinking it. Variations in eggs, vegetable oils, plant-based meat, milk and sugar substitutes’ consumption were not particularly noteworthy.

Multivariate logistic regression analysis suggested that BMI (overweight and obesity), education (university degree or higher), time since diagnosis (13-18 and 19-24 months), and type of breast cancer surgery (mastectomy), were significantly more likely associated with the change in consumption of baked goods and refined bread and pasta (**Table 3**). In particular, change in food consumption for factors category of overweight and obesity, higher education and time since diagnosis were mainly characterized by a decrease in baked good and refined bread and pasta consumption. Conversely, the multivariate model also significantly showed that increased age (mainly patients over 65 years) was less likely associated with a change in the consumption of these products (**Table 3**). Notably, multivariate analyses significantly showed that survivors older than 65 years were less likely to change all their food consumption habits, compared to the younger ones (**Supplementary Tables 2**–**12**), except for alcoholic drinks intake (**Supplementary Tables 13**). Other respondents’ demographic and clinical characteristics, associated or not with changes in food consumption are depicted in **Supplementary File 4** (**Supplementary Tables 2**–**13**).

**Table 3:** Factors associated with changes in baked goods, refined bread and pasta consumption.

Characteristics	Baked goods and refined bread and pasta		
	Multivariate analysis* (N = 393)		
	OR	95% CI	P-value
**BMI (Ref. Underweight or Normal weight)**			
Overweight or Obesity	2.20	1.33 – 3.64	**0.002**
**Age (Ref. <50)**			
50-64	0.50	0.25 – 0.97	**0.041**
>65	0.17	0.07 – 0.42	**<0.001**
**Education (Ref. Primary or middle** **school)**			
High school	1.42	0.86 – 2.34	0.173
University degree or higher	2.14	1.06 – 4.32	**0.033**
**Time since diagnosis, months (Ref. 1-6)**			
7-12	1.70	0.94 – 3.05	0.077
13-18	2.17	1.12 – 4.17	**0.021**
19-24	2.26	1.16 – 4.42	**0.017**
**Menopausal status (Ref. Pre)**			
Post	1.79	0.88 – 3.67	0.110
**Types of breast cancer surgery (Ref. Breast-conserving)**			
Mastectomy	1.77	1.08 – 2.90	**0.023**

N, number of respondents included in the regression.

*Model was adjusted for participating center.

OR, Odds Ratio; CI, Confidence Interval; BMI, Body Mass Index. In bold: p-value with a statistical significance level lower than 0.05.

### Nutrition Supplements Intake and Diets Followed, After BC Diagnosis

**Figure 2:**
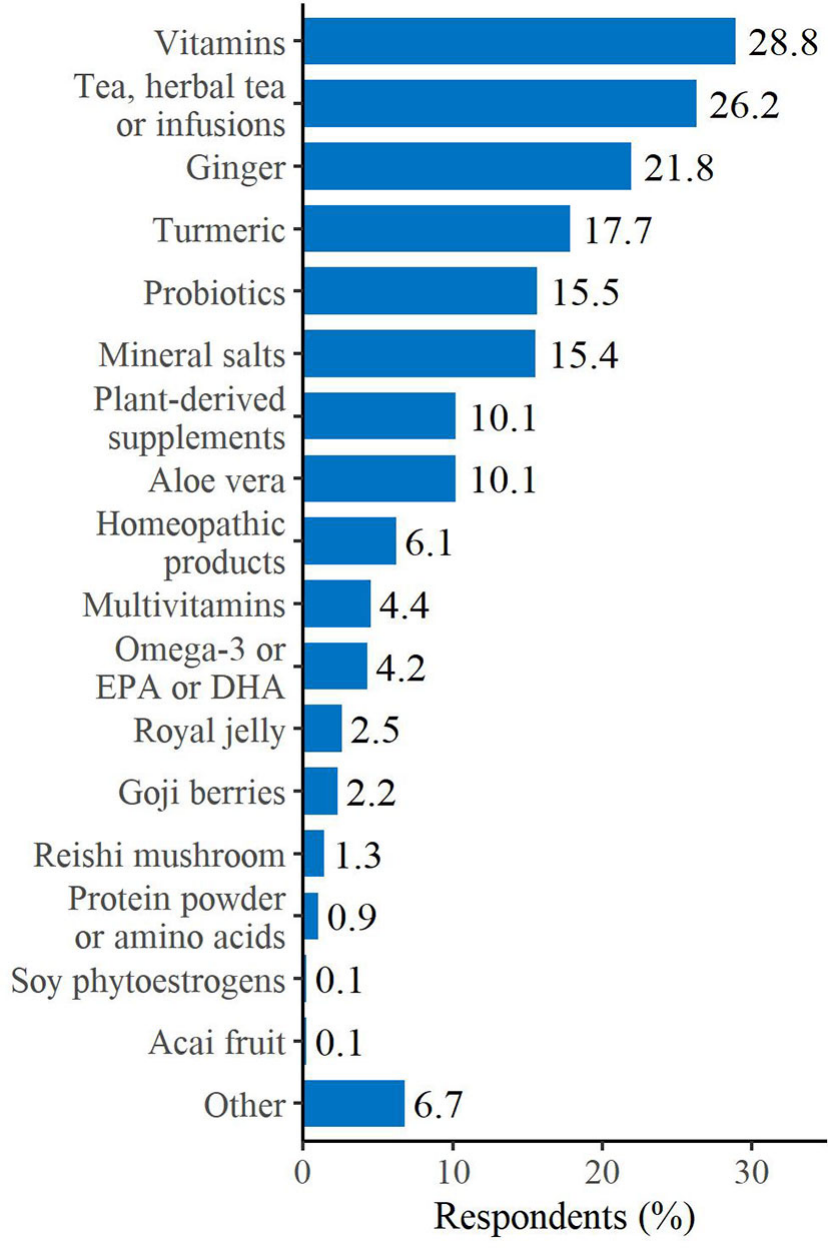
Supplements used after breast cancer diagnosis. Respondents could select more than one answer. “Other” includes: fruit extract/fruit juice, mushrooms, propolis/pollen and zeolite.

We also investigated whether our sample population of BC survivors started to use supplements or following specific diets, after cancer diagnosis, as frequently reported in the literature (29). Of note, the majority of patients reported having consumed, at least once, a nutritional supplement. These survivors mainly referred vitamins consumption (almost the 30%), followed by tea, herbal tea or infusions (26.2%) and ginger (21.8%). Probiotics and mineral salts were used by the 15.5% and the 15.4% of this sample, respectively. Homeopathic products were chosen only by the 6.1% of the survivors (**Figure 2**).

**Figure 3:**
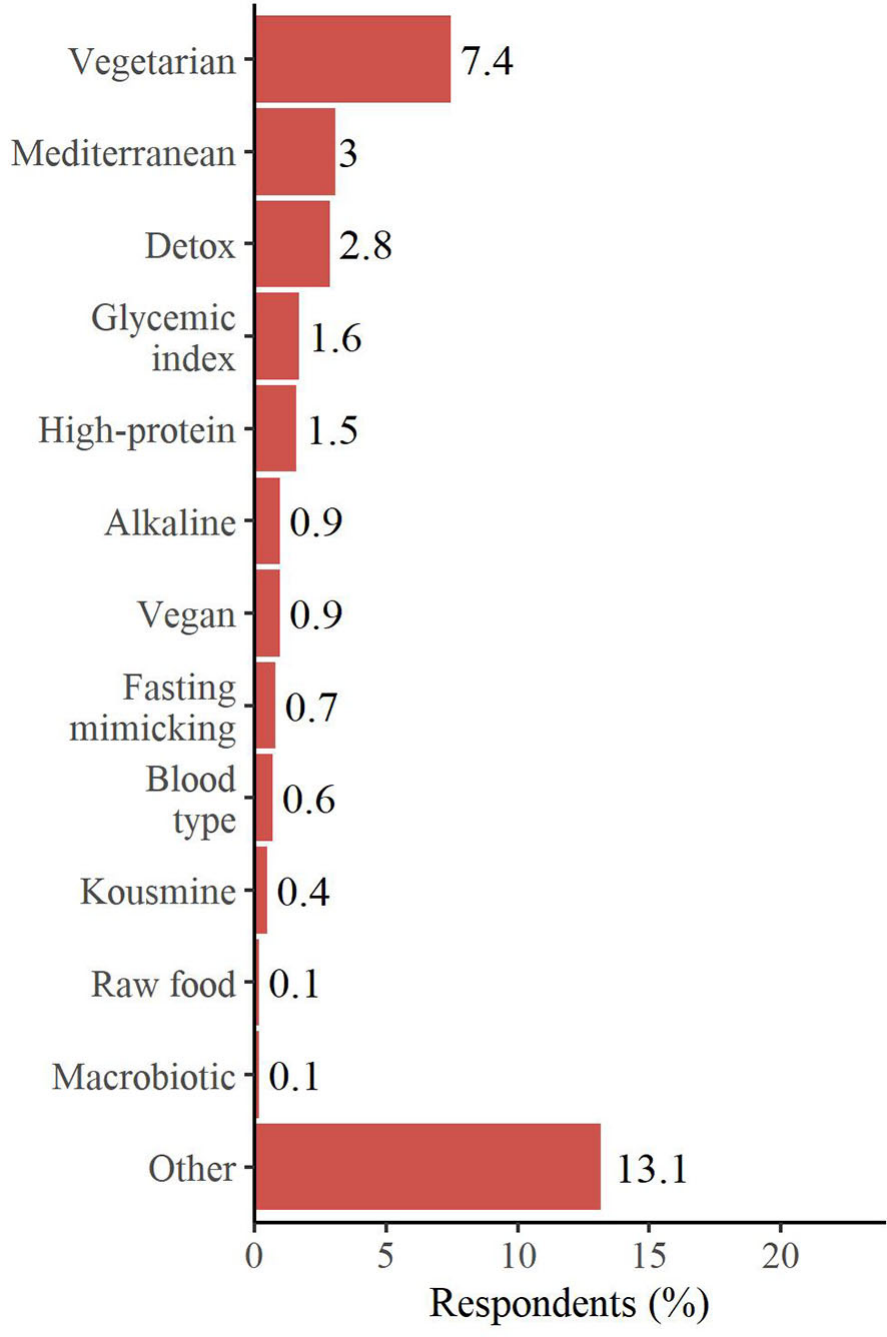
Diets started after breast cancer diagnosis. Respondents could select more than one answer. “Other” includes: balanced diet, diet suggested by the nutritionist, low-carb diet, DIANA (Diet and Androgens) diet (National Cancer Institute of Milan - INT), weight loss diet, hypocaloric diet, diet suggested by the dietician/other and low-fat diet.

After cancer diagnosis the majority of patients did not follow a specific diet, however, among the options chosen by the ones pursuing a particular nutritional pattern, the vegetarian diet stood out (7.4%), followed by the Mediterranean (3.0%) and the Detox diets (2.8%) (**Figure 3**).

As outlined in **Table 4**, we observed that the main reason, for BC survivors, to take supplements or start a specific diet was to counteract therapies’ side effects (31.6% and 11.5% of the sample, respectively). Patients did either consume supplements or follow a particular diet, regardless of their BMI status.

**Table 4:** Attitudes toward using nutritional supplements or following specific diets, after breast cancer diagnosis.

	Nutritional supplements		Diets	
	(N = 684)		(N = 684)	
	No.	(%)	No.	(%)
Reasons to take nutritional supplements or start specific diets*				
Counteract chemo/radiotherapy side effects	216	(31.6)	79	(11.5)
Nutritional deficiencies	70	(10.2)	74	(10.8)
Fight cancer	45	(6.6)	48	(7.0)
Lose weight	44	(6.4)	4	(0.6)
Other	122	(17.8)	40	(5.9)
Person who recommended taking nutritional supplements or starting specific diets*				
Myself through literature/Internet/Seminars	136	(19.9)	69	(10.1)
GP	118	(17.3)	68	(9.9)
Oncologist	95	(13.9)	41	(6.0)
Nutritionist	71	(10.4)	25	(3.7)
Pharmacist	54	(7.9)	23	(3.4)
Naturopathic or homeopathic doctor	52	(7.6)	17	(2.5)
Family or friends	40	(5.9)	14	(2.1)
Patients with the same medical condition	31	(4.5)	4	(0.6)
**Did you inform the oncologist about taking nutritional supplements or starting specific diets?**				
No	172	(25.1)	72	(10.5)
Yes	239	(34.9)	114	(16.7)
Missing	273	(39.9)	498	(72.8)

N, number of total respondents. Nutritional supplements: vitamins, tea, herbal tea or infusions, ginger, turmeric, probiotics, mineral salts, plant-derived supplements, aloe vera, homeopathic products, multivitamins, omega-3 or EPA or DHA, royal jelly, goji berries, reishi mushroom, protein powder or amino acids, soy phytoestrogens, acai fruit or other. Specific diets: vegetarian, detox, glycemic index, high-protein, vegan, alkaline, fasting mimicking, blood type, Kousmine, macrobiotic, paleo diet, raw food or other.

*Respondents could select more than one answer.

GP, General Practitioner.

Most of the patients, which started to consume nutritional supplements (19.9%) or following particular diets (10.1%), did not adhere to specific medical recommendation, but made those decisions relying on their own research from the Internet, books or seminars (**Table 4**). Moreover, the majority of them did not answered to the question asking whether they inform the oncologist about taking supplements or starting new diets (**Table 4**). However, the 34.9% of the survivors did acknowledge the oncologist about the supplements consumed and the diets followed (16.7%), while the remaining 25.1% and 10.5%, respectively, did not (**Table 4**).

### Patients Beliefs and Information Needs Regarding Nutrition and Cancer

The last 3 questions of the survey aimed to explore both the beliefs of BC survivors toward the association between nutrition and cancer and their need of information on this topic.

Interestingly, we discovered that 38.6% of patients think that a “moderate” link between cancer and nutrition does exist. Almost the same feeling was reported by the respondents aged below 65 years (41.0%); only the 31.4% of the patients over 65 years of age agreed with this answer, while the 19.2% of them believed that there is no connection between cancer and nutrition, compared to the 7.4% of the respondents aged below 65 years (**Table 5**). The major sources of information for the youngest survivors where the Internet, books or seminars (their own research) (28.2%), followed by the oncologist (27.7%) and the nutritionist (21.1%); while patients over 65 years of age mainly relied on the oncologist (13.5%) and general practitioner (12.2%) opinion. Only the 11.5% of them would use the Internet, books or seminars as sources of information (**Table 5**). Considering the overall sample, almost one fourth of the survivors either collected their information from the oncologist (24.4%) or from the Internet, books or seminars (24.0%) (**Table 5**).

**Table 5:** Beliefs toward cancer and nutrition.

	Total		Under 65 years		65 years or over		P°
	(N = 684)		(n = 517)		(n = 156)		
	No.	(%)	No.	(%)	No.	(%)	
**Do you agree that nutrition could be related to cancer?**							P<0.001
No	69	(10.1)	38	(7.4)	30	(19.2)	
A little	111	(16.2)	87	(16.8)	23	(14.7)	
Moderately	264	(38.6)	212	(41)	49	(31.4)	
A lot	112	(16.4)	95	(18.4)	16	(10.3)	
I do not know	105	(15.4)	72	(13.9)	30	(19.2)	
Missing	23	(3.4)	13	(2.5)	8	(5.1)	
After cancer diagnosis, where did you get nutrition information from?*							
My own research using books, the Internet and seminars	164	(24)	146	(28.2)	18	(11.5)	P<0.001
GP	86	(12.6)	65	(12.6)	19	(12.2)	P=1.000
Oncologist	167	(24.4)	143	(27.7)	21	(13.5)	P<0.001
Nutritionist	118	(17.3)	109	(21.1)	5	(3.2)	P<0.001
Pharmacist	10	(1.5)	8	(1.6)	2	(1.3)	P=1.000
Naturopath or homeopath	44	(6.4)	41	(7.9)	3	(1.9)	P=0.008
Relatives or friends	60	(8.8)	45	(8.7)	15	(9.6)	P=0.756
Other patients	80	(11.7)	71	(13.7)	9	(5.8)	P=0.008

**°**P-values were derived from the X² test for categorical data. N, number of total respondents.

GP, General Practitioner.

*Respondents could select more than one answer.

## Discussion

In this study, we aimed at exploring eating habits changes and supplement use in a group of Italian breast cancer survivors. Also, we wanted to identify the main sources of information accessed, which may have affected the referred changes and inspect whether those modifications have been reported to the physician.

Breast cancer survivors are known to describe positive changes in nutrition behavior after cancer diagnosis (29, 30, 33). In agreement with this, we observed that, after BC diagnosis, survivors made some changes, which respected, in part, the WCRF recommendations (5, 14, 15). This attitude has also been reported in a recent publication analyzing changes in dietary habits of an Italian group of patients, affected by different kind of tumors (31). Interestingly, authors described that almost the 60% of patients that referred a nutritional change were BC survivors (31). Consistently with the guidelines for cancer prevention and the recommendations for a healthy diet (5), our survivor population mainly described an increased consumption of vegetables, pulses, nuts, fresh fruit, wholemeal bread/pasta, grains and fresh fish. A rise in whole grain, fruit and vegetable consumption was also observed in a British cohort study, which analyzed a group of women, after 1 year of BC diagnosis (37). Similarly to that publication (37) and still consistently with the WCRF recommendations (5, 14, 15), a noteworthy decrease in the consumption of red and processed meat, refined bread/pasta, baked goods and animal fats was also observed. Differently from Velentzis L.S. et al. (37), despite our group of survivors reported a valuable decrease in soft drink consumption, they did not refer an important decline in alcoholic drink intake, whose consumption is indeed strongly associated with an increase in breast cancer risk (5, 14, 15). Many misconceptions about diet and cancer do exist, including the belief, very often reported on the Internet, of milk being able to increase the risk of breast cancer development and recurrence. Regardless of that, our BC survivor group did not state a noteworthy variation in milk consumption.

Despite the abovementioned changes, diet modifications were mostly pursued by less than half of the overall sample; moreover, a trend toward diminishing the intake of many food items was detected (red and processed meat, refined bread/pasta, baked goods and animal fats), more than an increase in the consumption of healthy foods recommended for the general cancer prevention (vegetables, pulses, nuts, fresh fruit, wholemeal bread/pasta, grains and fresh fish). We hypothesized that this limitation in food intake could be related to the willing of maintaining or reaching a healthy body weight, considering that many BC survivors gain weight following treatment (38). For instance, even though the limitation in carbohydrate intake is not necessarily the healthiest way to lose weight, many people consider this practice a healthful method to get back in shape. Remarkably, change in food intake for the factor category “overweight and obesity” was mainly characterized by a decrease in baked good and refined bread and pasta consumption.

Of note, we observed that survivors older than 65 years were less likely to change their food consumption, compared to younger patients. It is unlikely that this lack of change could be explained with a high frequency of healthy food behaviors before diagnosis, considering that the proportion of overweight or obese survivors in this group is statistically significantly higher, compared to the younger patients. Possibly, this group of patients might stay more attached to their food habits, hence be less likely to modify dietary behaviors. These findings suggest the need to primarily target specific nutritional intervention toward older BC survivors, as they may experience more difficulty to initiate and/or maintain change on their own.

Next, we analyzed whether this population of BC survivors started to use supplements or following particular diets, after cancer diagnosis, as commonly described in the literature (29–31, 37, 39, 40). In agreement with previous publications (31, 37, 39, 40), more than 50% of the patients reported having consumed, at least once, a nutritional supplement, mainly referring vitamins intake and, even if at a lower percentage, mineral salts and omega-3/fish oil. Survivors stated that the main reason to take supplements was to counteract therapies’ side effects, as previously reported (39). In contrast to supplement consumption, the majority of patients chose not to follow a specific diet, after BC diagnosis. The few who decided to pursue a particular nutritional pattern predominantly opted for the vegetarian diet, followed by the Mediterranean and the Detox diets. Remarkably, CUP Expert Panel of WCRF/AICR showed strong evidence that consuming a “Mediterranean type” dietary pattern decreases the risk of weight gain, overweight and obesity, thus indirectly protecting against breast cancer risk (8). Moreover, mounting evidence finds inverse association between Mediterranean Diet (MD) adherence and both receptor negative and triple-negative BC incidence (41–43).

The BC-preventive effect of MD is possibly due to its peculiar combination of foods rich in anti-oxidants and anti-inflammatory bioactive nutrients (phenolic compounds, omega-3 polyunsaturated fatty acids, retinoids, etc.), which are believed to exert a protective role against cancer development and progression, preventing DNA damage and reducing cell degeneration, proliferation and metastasis (44). As for supplements, patients started to follow specific diets mainly with the aim of controlling treatment side effects; in agreement with this, no correlation between BMI higher than 25 and beginning a new diet was observed.

As already reported (30), most of the patients that started to consume nutritional supplements or following a diet had not received professional advice about it; instead, they mainly sought information from the Internet, books or seminars. Moreover, only less than 35% and 17% of survivors, respectively, did acknowledge the oncologist about taking supplements or beginning new diets. Taking all this into account, it might be advisable for clinicians to regularly ask patients about their supplement use and suggest caution due to a lack of evidence of any beneficial effects and, instead, of potential increased risks, in term of cancer recurrence and harmful interaction with therapies (39). Besides, oncologists and other healthcare professionals should be receptive to questions about that topic and prepared to guide patients toward a proper use of dietary supplements.

Interestingly, when survivors were asked about their beliefs toward the association between nutrition and cancer, around the 40% of them thinks that a moderate link between cancer and nutrition does exist; however, almost one fifth of patients over 65 years of age believed that there is no connection between cancer and nutrition. Considering the overall sample, the sources of information mostly used to gain insight on tumor risk and nutrition, were, at the same proportion, the oncologist and Internet/books/seminars. The latter were mostly consulted by younger patients, while survivors older than 65 years mainly relied on physician opinion (oncologists and general practitioners).

Overall, data from our survey indicate that, despite BC survivors were aware of a few dietary messages and made some positive change in their nutritional habits, those modifications were not always adherent to the guidelines for cancer prevention. Also, survivors older than 65 years were remarkably less motivated to modify their food consumption, compared to younger patients. Moreover, we observed that the majority of patients consumed nutritional supplements, after diagnosis, without having previously consulted or informed the oncologist. Considering also that a significant number of BC survivors searched for information about the link between tumor and nutrition in general media or online, it is possible that they followed messages, deemed to be reliable and accurate, which were, instead, incorrect or misleading. Some papers have shown that cancer survivors eager to obtain information on cancer and nutrition, and they often recur to Internet search because they encounter difficulties in seeking nutrition advice from healthcare providers (45, 46). Accordingly, it is imperative to ameliorate and strengthen the physician-patient relationship, in order to allow health professionals to better intercept survivor needs, thus providing tailored nutrition counselling and lifestyle intervention programs across the cancer continuum.

Two main strengths of this study include the use of a pre-tested questionnaire and the survey administration method. The employment of a tested survey decreases sampling error and increases the final questionnaire response rates. On the other hand, self-reported data have been shown to reduce the interviewer bias and possibly promote truthful responses. Moreover, our questionnaire gives the opportunity to perform a descriptive analysis of the hypothetical correlations existing among clinical and biopathological characteristics of the tumor, pharmacological treatments and dietary changes, as described in the “Patients and methods” section.

However, some limitations need to be considered. Due to the self-reported nature of the survey, patients may inaccurately recall their nutrition habits and/or be inclined to over emphasize their food intakes towards healthier choices, since this appears more socially desirable. Also, we used a qualitative approach to explore the eating behaviors, thus we lack a quantitative measurement of food consumption. Finally, we did not analyze and compare dietary habits, before and after diagnosis.

## Conclusion

Importantly, our findings contribute to further understanding BC survivors’ dietary needs/behaviors and identifying certain patient categories that can possibly represent critical primary target for tailored lifestyle interventions.

Moreover, this study showed, once again, the need for developing and implementing lifestyle recommendations for cancer survivors and integrating nutrition guidance into oncology care. Accordingly, it is fundamental to provide training and continuing education opportunities to health professionals, so that they can play a larger role in offering appropriate nutritional guidance, counselling and nutrition education to survivors and caregivers. National health service should encourage patients and caregivers not only to follow the conventional nutritional guidelines and join cancer survivor groups, but also to use customized mobile app, designed by experts, to guide them towards optimal nutrition and lifestyle choices.

The ECHO Survey offers the opportunity to be applied to bigger group of BC survivors, in order to collect more information and eventually improve the ongoing intervention programs. Moreover, this questionnaire can be administered to people with other cancer types, starting from prostate and colorectal tumors, which both show a very high incidence and prevalence worldwide. This would help to further expand the existing knowledge on patients’ information needs, dietary behaviors and beliefs about nutrition, thus promoting adherence to optimal personalized lifestyle recommendations aimed at preventing tumor recurrence and increasing survival rates.

## Data Availability Statement

The data supporting the conclusions of this article will be made available by the authors, without undue reservation.

## Ethics Statement

Ethics approval was obtained from the Ethical Committee for Clinical Trials of the Provinces of Verona and Rovigo and from the Local Ethic Committees of the other collaborating oncology centers. Informed consent and patient details are described in the “Material and methods” section. The patients/participants provided their written informed consent to participate in this study.

## Author Contributions

GC contributed to data analysis, results discussion and interpretation; she prepared and wrote the manuscript. MaT contributed to the experimental design, data analysis, results discussion and interpretation. AF, VG, CF, MVD, BB, AB, SC, PV, EM, CZ administered and collected the questionnaire, contributed to results discussion and interpretation. MoT, AM, FM, MV, FN contributed to results discussion and interpretation. CM performed the statistical analysis, contributed to data analysis, results discussion and interpretation. FG contributed to the experimental design. LT contributed to the experimental design, results discussion and interpretation. SG administered and collected the questionnaire, contributed to results discussion and interpretation and she supervised the work. All authors read the manuscript and approved the submitted version.

## Conflict of Interest

The authors declare that the research was conducted in the absence of any commercial or financial relationships that could be construed as a potential conflict of interest.

## Publisher’s Note

All claims expressed in this article are solely those of the authors and do not necessarily represent those of their affiliated organizations, or those of the publisher, the editors and the reviewers. Any product that may be evaluated in this article, or claim that may be made by its manufacturer, is not guaranteed or endorsed by the publisher.
