# Corrigendum: Results of the ECHO (Eating habits CHanges in Oncologic patients) Survey: An Italian Cross-Sectional Multicentric Study to Explore Dietary Changes and Dietary Supplement Use, in Breast Cancer Survivors

**DOI:** 10.3389/fonc.2022.851999

**Published:** 2022-02-02

**Authors:** Greta Caprara, Maria Tieri, Alessandra Fabi, Valentina Guarneri, Cristina Falci, Maria Vittoria Dieci, Monica Turazza, Bettina Ballardini, Alessandra Bin, Saverio Cinieri, Patrizia Vici, Emilia Montagna, Claudio Zamagni, Cristina Mazzi, Alessandra Modena, Fabiana Marchetti, Matteo Verzè, Francesca Ghelfi, Lucilla Titta, Fabrizio Nicolis, Stefania Gori

**Affiliations:** ^1^Department of Experimental Oncology, Istituto Europeo di Oncologia (IEO), European Institute of Oncology, Istituto di Ricovero e Cura a Carattere Scientifico (IRCCS), Milano, Italy; ^2^Fondazione Tera, Novara, Italy; ^3^ Medical Oncology 1 – Istituto Nazionale Tumori Regina Elena Istituto di Ricovero e Cura a Carattere Scientifico (IRCCS), Roma, Italy; ^4^Precision Medicine in Breast Cancer Unit, Fondazione Policlinico Universitario A. Gemelli Istituto di Ricovero e Cura a Carattere Scientifico (IRCCS), Roma, Italy; ^5^Department of Surgery, Oncology and Gastroenterology, University of Padova, Padova, Italy; ^6^Medical Oncology 2 - Istituto Oncologico Veneto Istituto Oncologico Veneto (IOV) Istituto di Ricovero e Cura a Carattere Scientifico (IRCCS), Padova, Italy; ^7^Department of Oncology, Istituto di Ricovero e Cura a Carattere Scientifico (IRCCS) Sacro Cuore Don Calabria Hospital, Negrar di Valpolicella, Italy; ^8^Breast Division, MultiMedica Breast Unit Multimedica Istituto di Ricovero e Cura a Carattere Scientifico (IRCCS), Milano, Italy; ^9^Dipartimento di Oncologia, Azienda Sanitaria Universitaria Integrata di Udine, Udine, Italy; ^10^Unità Operativa Complessa di Oncologia Medica, ASL Brindisi Senatore Antonio Perrino Hospital, Brindisi, Italy; ^11^Unità Operativa Semplice Dipartimentale (UOSD), Sperimentazioni di FASE IV, Istituto Nazionale Tumori Regina Elena Istituto di Ricovero e Cura a Carattere Scientifico (IRCCS), Roma, Italy; ^12^Division of Medical Senology, Istituto Europeo di Oncologia (IEO), European Institute of Oncology, Istituto di Ricovero e Cura a Carattere Scientifico (IRCCS), Milano, Italy; ^13^Medical Oncology Unit, Istituto di Ricovero e Cura a Carattere Scientifico (IRCCS) Azienda Ospedaliero-universitaria di Bologna, Bologna, Italy; ^14^Clinical Research Unit, Istituto di Ricovero e Cura a Carattere Scientifico (IRCCS) Sacro Cuore Don Calabria Hospital, Negrar di Valpolicella, Italy; ^15^Medical Direction, Istituto di Ricovero e Cura a Carattere Scientifico (IRCCS) Sacro Cuore Don Calabria Hospital, Negrar di Valpolicella, Italy; ^16^Fondazione De Marchi-Department of Pediatrics, Istituto di Ricovero e Cura a Carattere Scientifico (IRCCS) Ca’ Granda Ospedale Maggiore Policlinico, Milano, Italy; ^17^The Need For Nutrition Education/Innovation Programme (NNEdPro) Global Centre for Nutrition and Health, St John’s Innovation Centre, Cambridge, United Kingdom; ^18^Associazione Italiana di Oncologia Medica (AIOM) Foundation Past President, Medical Direction, Istituto di Ricovero e Cura a Carattere Scientifico (IRCCS) Sacro Cuore Don Calabria Hospital, Negrar di Valpolicella, Italy; ^19^Associazione Italiana di Oncologia Medica (AIOM) Foundation President, Department of Oncology, Istituto di Ricovero e Cura a Carattere Scientifico (IRCCS) Sacro Cuore Don Calabria Hospital, Negrar di Valpolicella, Italy

**Keywords:** eating habits survey, health information, dietary changes, cancer patients, cancer survivors, breast cancer

In the published article, there was an error regarding the affiliation for Maria Vittoria Dieci. As well as having affiliation 6, she should also have affiliation 5.

In the published article there was an error in affiliation 4. Instead of “Breast Precision Medicine Unit, Policlinico Universitario A. Gemelli Istituto di Ricovero e Cura a Carattere Scientifico (IRCCS), Roma, Italy”, it should be “Precision Medicine in Breast Cancer Unit, Fondazione Policlinico Universitario A. Gemelli Istituto di Ricovero e Cura a Carattere Scientifico (IRCCS), Roma, Italy.”

Moreover, In the published article there was an error in affiliation 11. Instead of “Medical Oncology 2 - Istituto Nazionale Tumori Regina Elena Istituto di Ricovero e Cura a Carattere Scientifico (IRCCS), Roma, Italy”, it should be “Unità Operativa Semplice Dipartimentale (UOSD), Sperimentazioni di FASE IV, Istituto Nazionale Tumori Regina Elena Istituto di Ricovero e Cura a Carattere Scientifico (IRCCS), Roma, Italy.”

The authors apologize for these errors and state that this does not change the scientific conclusions of the article in any way.

## Publisher’s Note

All claims expressed in this article are solely those of the authors and do not necessarily represent those of their affiliated organizations, or those of the publisher, the editors and the reviewers. Any product that may be evaluated in this article, or claim that may be made by its manufacturer, is not guaranteed or endorsed by the publisher.

