# Supplementary material for: Results of the ECHO (Eating habits CHanges in Oncologic patients) Survey: An Italian Cross-Sectional Multicentric Study to Explore Dietary Changes and Dietary Supplement Use, in Breast Cancer Survivors
Source: Front Oncol. 2021 Nov 1;11:705927. doi: 10.3389/fonc.2021.705927 (PMC8596328; doi:10.3389/fonc.2021.705927)
Supplement: Supplementary file 1 [file DataSheet_1.docx]

Supplementary Material

# Supplementary Data

**SUPPLEMENTARY FILE 1**. Oncologist form.

**PERSPECTIVE OBSERVATIONAL STUDY ON CHANGES IN DIETARY HABITS AFTER BREAST CANCER DIAGNOSIS (ECHO STUDY)**

CENTER CODE #______

PATIENT CODE #______

PATIENT SUBGROUP:

A B C D Date of form completion___/___/_____

Date of birth___/___/_____

**Menopausal status at diagnosis:** ☐ **PRE** ☐ **POST**

Weight: (kg)____Height: (cm)____PS (ECOG)____

Date of first diagnosis (cytology/histology)___/___/_____

**Neoadjuvant treatment:** ☐ **NO** ☐ **YES** If YES: Start Date ___/___/_____

**Type**: ☐ Hormone therapy ☐ Chemotherapy ☐ Chemotherapy + Trastuzumab ☐ Other (specify)______________________

**Clinical Status: cT___________ cN___________ M0___________**

**Histotype:** ☐ Invasive ductal carcinoma ☐ Invasive lobular carcinoma ☐ Other (specify)_________________________

**Grading:** ☐ G1 ☐ G2 ☐ G3 ☐ Unknown

**Hormone receptors (ER and/or PGR):** ☐ Positive ☐ Negative ☐ Unknown

**HER2:** ☐ Positive ☐ Negative ☐ Unknown; **Ki67 (o MIB1):** specify percentage_____% ☐ Unknown

**Surgery**

Breast surgery: ☐ **NO** ☐**YES;** If **YES**: **date**__/___/____**; Type:** ☐ Breast-conserving ☐ Mastectomy

Axillary surgery: ☐ **NO** ☐ **YES;** If **YES**: **date**__/__/____; **Type**: ☐ Sentinel node biopsy ☐ Axillary dissection

**Post-operative biological and molecular parameter status:**

**Histotype:** ☐ Invasive ductal carcinoma ☐ Invasive lobular carcinoma ☐ Other (specify)_________________________

**Grading:** ☐ G1 ☐ G2 ☐ G3 ☐ Unknown

**Hormone receptors (ER and/or PGR):** ☐ Positive ☐ Negative ☐ Unknown

**HER2:** ☐ Positive ☐ Negative ☐ Unknown; **Ki67 (o MIB1):** specify percentage_______% ☐ Unknown

**Pathological status:** ☐ **IA** ☐ **IB** ☐ **IIA** ☐ **IIB** ☐ **IIIA** ☐ **IIIB** ☐ **IIIC**

**BRCA1:** ☐ Mutated ☐ Not mutated ☐ Unknown ☐ Evaluation in progress

**BRCA2**: ☐ Mutated ☐ Not mutated ☐ Unknown ☐ Evaluation in progress

Oncologist Form V. 1 dated 26/07/2017 Page 1

**Adjuvant treatment:** ☐ **NO** ☐ **YES** If YES: Start Date___/___/_____

**Type**: ☐ Hormone therapy ☐ Chemotherapy + Hormone therapy ☐ Chemotherapy + Hormone therapy + Trastuzumab

☐ Other (specify)___________

**Chemotherapy**: ☐ Anthracycline ☐ Taxanes ☐ Anthracycline + Taxanes ☐ Other (specify)________

**Trastuzumab**: ☐ No ☐ Yes

**Hormone therapy**: ☐ TAM ☐ TAM + LHRH ☐ AI + LHRH ☐ AI ☐ Other (specify)___________

**Post-operative radiotherapy** ☐ NO ☐ YES

**Outcome: Local-regional recurrence:** ☐ NO ☐ YES; **If YES**: Date of diagnosis___/___/________

**Area of recurrence**: ☐ Local-regional ☐ Distance metastatic recurrence

Oncologist Form V. 1 dated 26/07/2017 Page 2

**SUPPLEMENTARY FILE 2**. The “ECHO” (Eating habits CHanges in Oncologic patients) questionnaire.

CENTER CODE #______

PATIENT CODE #______

PATIENT SUBGROUP:

A B C D Date_____________

# ‘ECHO’ QUESTIONNAIRE

Dear patient,

With the following questionnaire, we aim to add to our current data so as to more accurately identify changes in patients’ dietary habits, following a cancer diagnosis. Our intention is to evaluate how frequently, and to what degree, changes in diet occur and to identify the information sources most often used.

The questionnaire is simple to complete and will only take a few minutes of your time. It is completely anonymous.

How to fill in the form:

Answer each question in the questionnaire by ticking the relevant answer. If you are uncertain, choose the answer that fits best.

Please tick one answer only out of those listed, unless the question itself allows for more than one option (*“more than one answer possible”*).

Bear in mind that this questionnaire is not an evaluation and there are no right or wrong answers.

We ask you to answer the questions personally. Please do not get your partner, relatives, friends or visitors to help you in the completion of this questionnaire.

Thank you for taking the time to complete this questionnaire for us.

**ECHO QUESTIONNAIRE**

| **PERSONAL DETAILS AND CANCER THERAPIES** |
| --- |

| 1. Age |  | 〇18-34 years  〇35-49 years  〇50-64 years  〇65-79 years  〇 > 80 years |
| --- | --- | --- |
| 2. Gender |  | 〇 F  〇 M |
| 3. Level of Education |  | 〇 Primary/Middle School  〇 High School  〇 University Degree  〇 Master’s/PhD |
| 4. Cancer treatments – finished and/or ongoing  *(****more than one answer possible****)* |  | ☐ None  ☐ Surgery  ☐ Radiotherapy  ☐ Chemotherapy  ☐ Hormone therapy  ☐ Biological therapy |

| **VITAMINS, OTHER SUPPLEMENTS AND SPECIFIC DIETS AFTER A DIAGNOSIS OF CANCER** |
| --- |

| 5. Did you begin to use one or more of these products following your diagnosis? *(****more than one answer possible****)* | ☐ vitamins (eg vit. C, vit. D)  ☐ mineral salts (eg calcium, potassium, magnesium)  ☐ multivitamins  ☐ omega-3, EPA/DHA  ☐ plant-derived supplements (tablets/capsules/drops)  ☐ lactic cultures/probiotics  ☐ homeopathic products  ☐ soy phytoestrogens  ☐ protein powder/amino acids  ☐ tea/herbal tea/infusions  ☐ royal jelly  ☐ aloe vera  ☐ reishi mushroom  ☐ açai fruit  ☐ goji berries  ☐ turmeric  ☐ ginger  ☐ other *(please specify)*_________________ |
| --- | --- |
| 5.1. If your answer was YES, why have you used these products?  (***more than one answer possible)*** | ☐ a nutritional deficiency  ☐ help to lose weight  ☐ help during chemo/radiotherapy  ☐ to fight cancer  ☐ other *(please specify)*_________________ |
| 5.2. If your answer was YES, who suggested the use of these products or gave you information about them?  *(****more than one answer possible****)* | ☐ myself using literature/Internet/ seminars  ☐ general practitioner (GP)  ☐ oncologist  ☐ dietician/nutritionist  ☐ pharmacist  ☐ naturopathic doctor/homeopathic doctor/other non-conventional medical practitioner  ☐ family/friend  ☐ patients with the same medical condition |
| 5.3. If your answer was YES, have you spoken with your oncologist about the products you take? | 〇 Yes  〇 No |

| 6. If you started a specific diet after your diagnosis, which type of diet was it?  *(****more than one answer is possible****)* | ☐ vegetarian diet  ☐ vegan diet  ☐ macrobiotic diet  ☐ high-protein diet  ☐ blood type diet  ☐ alkaline diet  ☐ glycemic index diet  ☐ paleo diet  ☐ raw food diet  ☐ detox diet/based on fruit and/or vegetable juices  ☐ Kousmine method diet  ☐ fasting mimicking diet  ☐ other *(please specify)*_________________ |
| --- | --- |
| 6.1. If you started a specific diet, what were your reasons for doing so?  *(****more than one answer is possible****)* | ☐ a nutritional deficiency  ☐ help to lose weight  ☐ help during chemo/radiotherapy  ☐ to fight cancer  ☐ other *(please specify)*_________________ |
| 6.2. If you started a specific diet, who suggested or gave you information about the diet? *(****more than one answer possible****)* | ☐ myself using literature/Internet/ seminars  ☐ general practitioner (GP)  ☐ oncologist  ☐ dietician/ nutritionist  ☐ pharmacist  ☐ naturopathic doctor/homeopathic doctor/other non-conventional medical practitioner  ☐ family or friends  ☐ patients with same medical condition |
| 6.3. If you started a specific diet, have you spoken with your oncologist about the diet you started? | 〇 Yes  〇 No |

| **EATING HABITS AFTER CANCER DIAGNOSIS** |
| --- |

| ***CEREALS AND CEREAL PRODUCTS***  *How have your eating habits changed* ***since your diagnosis****?*  *(Only one answer is possible for each question)*  **7. WHOLEMEAL BREAD AND PASTA 8. REFINED BREAD AND PASTA**  〇 eat the same 〇 eat the same  〇 have started eating 〇 have started eating  〇 eat more 〇 eat more  〇 eat less 〇 eat less  〇 no longer eat 〇 no longer eat  〇 have never eaten 〇 have never eaten  **9. BAKED GOODS 10. GRAINS**  **(crackers, breadsticks, biscuits, cakes, etc.) (rice, spelt, barley, quinoa,**  **buckwheat, amaranth, etc.)**  〇 eat the same 〇 eat the same  〇 have started eating 〇 have started eating  〇 eat more 〇 eat more  〇 eat less 〇 eat less  〇 no longer eat 〇 no longer eat  〇 have never eaten 〇 have never eaten |
| --- |

| **EATING HABITS AFTER CANCER DIAGNOSIS** |
| --- |

| ***FRUIT, VEGETABLES, PULSES***  *How have your eating habits changed* ***since your diagnosis****?*  *(Only one answer is possible for each question)*  **11. FRESH FRUIT 12. NUTS**  **(walnuts, hazelnuts, almonds, pistachios, etc.)**  〇 eat the same 〇 eat the same  〇 have started eating 〇 have started eating  〇 eat more 〇 eat more  〇 eat less 〇 eat less  〇 no longer eat 〇 no longer eat  〇 have never eaten 〇 have never eaten  **13. VEGETABLES 14. PULSES**  **(salads, cooked vegetables, vegetable soups, etc.) (chickpeas, beans, lentils, fava beans, peas, etc.)**  〇 eat the same 〇 eat the same  〇 have started eating 〇 have started eating  〇 eat more 〇 eat more  〇 eat less 〇 eat less  〇 no longer eat 〇 no longer eat  〇 have never eaten 〇 have never eaten |
| --- |

| **EATING HABITS AFTER CANCER DIAGNOSIS** |
| --- |

| ***MEAT, MEAT PRODUCTS AND MEAT SUBSTITUTES***  *How have your eating habits changed* ***since your diagnosis****?*  *(Only one answer is possible for each question)*  **15. RED MEAT 16. WHITE MEAT**  **(beef, pork, lamb) (chicken, turkey, rabbit)**  〇 eat the same 〇 eat the same  〇 have started eating 〇 have started eating  〇 eat more 〇 eat more  〇 eat less 〇 eat less  〇 no longer eat 〇 no longer eat  〇 have never eaten 〇 have never eaten  **17. PROCESSED MEAT 18. PLANT-BASED MEAT SUBSTITUTES**  **(cold meats, salami, cured meats, hot dogs, etc.) (soya burgers, seitan, vegetable nuggets, etc.)**  〇 eat the same 〇 eat the same  〇 have started eating 〇 have started eating  〇 eat more 〇 eat more  〇 eat less 〇 eat less  〇 no longer eat 〇 no longer eat  〇 have never eaten 〇 have never eaten |
| --- |

| ***FISH***  *How have your eating habits changed* ***since your diagnosis****?*  *(Only one answer is possible for each question)*  **19. FISH AND SHELLFISH 20. PRESERVED FISH**  **(fresh, frozen) (canned, smoked)**  〇 eat the same 〇 eat the same  〇 have started eating 〇 have started eating  〇 eat more 〇 eat more  〇 eat less 〇 eat less  〇 no longer eat 〇 no longer eat  〇 have never eaten 〇 have never eaten |
| --- |

| **EATING HABITS AFTER CANCER DIAGNOSIS** |
| --- |

| ***MILK AND MILK SUBSTITUTES***  *How have your eating habits changed* ***since your diagnosis****?*  *(Only one answer is possible for each question)*  **21. MILK 22. PLANT-BASED MILK SUBSTITUTES**  **(UHT, fresh, full-cream, semi-/skimmed, lactose-free) (soya, rice, oat, kamut, etc.)**  〇 eat the same 〇 eat the same  〇 have started eating 〇 have started eating  〇 eat more 〇 eat more  〇 eat less 〇 eat less  〇 no longer eat 〇 no longer eat  〇 have never eaten 〇 have never eaten |
| --- |

| ***CHEESE AND EGGS***  *How have your eating habits changed* ***since your diagnosis****?*  *(Only one answer is possible for each question)*  **23. CHEESE 24. EGGS**  **(fresh, mature, grated, spread, low-fat, etc.)**  〇 eat the same 〇 eat the same  〇 have started eating 〇 have started eating  〇 eat more 〇 eat more  〇 eat less 〇 eat less  〇 no longer eat 〇 no longer eat  〇 have never eaten 〇 have never eaten |
| --- |

| ***DRESSINGS AND CONDIMENTS***  *How have your habits changed* ***since your diagnosis****?*  *(Only one answer is possible for each question)*  **25. VEGETABLE OILS 26. ANIMAL FATS**  **(extra-virgin olive oil, seed oil) (butter, lard, cream)**  〇 eat the same 〇 eat the same  〇 have started eating 〇 have started eating  〇 eat more 〇 eat more  〇 eat less 〇 eat less  〇 no longer eat 〇 no longer eat  〇 have never eaten 〇 have never eaten |
| --- |

| **EATING HABITS AFTER CANCER DIAGNOSIS** |
| --- |

| ***DRINKS***  *How have your habits changed* ***since your diagnosis****?*  *(Only one answer is possible for each question)*  **27. ALCOHOLIC DRINKS 28. SOFT DRINKS**  **(wine, beer, spirits, liqueurs, cocktails, etc.) (fruit juice, iced tea, cola, lemonade, tonic water, etc.)**  〇 eat the same 〇 eat the same  〇 have started eating 〇 have started eating  〇 eat more 〇 eat more  〇 eat less 〇 eat less  〇 no longer eat 〇 no longer eat  〇 have never eaten 〇 have never eaten |
| --- |

| ***SWEETS AND DESSERTS***  *How have your eating habits changed* ***since your diagnosis****?*  *(Only one answer is possible for each question)*  **29. HOMEMADE CAKES AND DESSERTS 30. SUGAR SUBSTITUTES**  **(tarts, cakes, pastries, ice cream, desserts etc.) (agave syrup, barley or rice malt, stevia,**  **other sweeteners, etc.)**  〇 eat the same 〇 eat the same  〇 have started eating 〇 have started eating  〇 eat more 〇 eat more  〇 eat less 〇 eat less  〇 no longer eat 〇 no longer eat  〇 have never eaten 〇 have never eaten |
| --- |

| **FOOD AND NUTRITION: PERSONAL OPINIONS AND SOURCES OF INFORMATION** |
| --- |

| 31. Do you agree that food and nutrition could be linked to cancer? | 〇 no  〇 not much  〇 fairly  〇 very  〇 I don’t know |
| --- | --- |
| 32. After your diagnosis did you ask anyone for information on nutrition? | 〇 YES  〇 NO |
| 32.1. If your answer was YES, who did you go to for this information?  *(****more than one answer is possible****)* | ☐ my own research using literature /Internet/seminars  ☐ general practitioner (GP)  ☐ oncologist  ☐ dietician/nutritionist  ☐ pharmacist  ☐ naturopathic doctor/homeopathic doctor/other non-conventional medical practitioner  ☐ family/friends  ☐ patients with same medical condition |

# Supplementary Tables

**SUPPLEMENTARY FILE 3.** Patient distribution among the participating oncology centers.

**SUPPLEMENARY TABLE 1**. Patient sample (N = 684) distribution among the different participating oncology centers.

|  | **Patients (N = 684)** | |
| --- | --- | --- |
| **Participating oncology centers*** | **No.** | **(%)** |
| Sacro Cuore Don Calabria Hospital, Verona | 101 | (14.8) |
| Istituto Oncologico Veneto, Padova | 106 | (15.5) |
| Senatore Antonio Perrino Hospital, Brindisi | 74 | (10.8) |
| INT - Regina Elena, Medical Oncology 1, Roma | 132 | (19.3) |
| INT - Regina Elena, Medical Oncology 2, Roma | 39 | (5.7) |
| Azienda Sanitaria Universitaria Integrata, Udine | 77 | (11.3) |
| Istituto Europeo di Oncologia, Milano | 36 | (5.3) |
| Breast Unit Multimedica, Milano | 91 | (13.3) |
| Azienda ospedaliero-universitaria  Policlinico Sant’Orsola-Malpighi,  Bologna | 28 | (4.1) |
| NOTE. N: number of total respondents.  *Detailed center addresses: Medical Oncology, IRCCS Sacro Cuore Don Calabria Hospital, Negrar di Valpolicella, Verona, Italy; Oncologia Medica 2 Istituto Oncologico Veneto, IOV, IRCCS, Padova, Italy; ASL Brindisi Senatore Antonio Perrino Hospital, Brindisi, Italy; Oncologia Medica 1 - Istituto Nazionale Tumori, INT, Regina Elena, Roma, Italy; Oncologia Medica 2 - Istituto Nazionale Tumori Regina Elena, Roma, Italy; Azienda Sanitaria Universitaria Integrata di Udine, Italy; Istituto Europeo di Oncologia, IEO, Milano, Italy; Breast Unit Multimedica, Milano, Italy; Azienda ospedaliero-universitaria Policlinico Sant’Orsola-Malpighi, Bologna, Italy. | | |

**SUPPLEMENTARY FILE 4.** Results of the univariate and multivariate analyses of the changes in food consumption.

SUPPLEMENTARY TABLE 2. Factors associated with the change in red and processed meat consumption.

|  | **Red and processed meat** | | | | | |
| --- | --- | --- | --- | --- | --- | --- |
|  | **Univariate analysis (N=402)** | | | **Multivariate analysis* (N=402)** | | |
| **Characteristics** | **OR** | **95% CI** | **P-value** | **OR** | **95% CI** | **P-value** |
| **BMI (Ref. Underweight or Normal**  **weight)** |  |  |  |  |  |  |
| Overweight or Obesity | 1.18 | 0.77, 1.80 | 0.449 |  |  |  |
| **Age, years (Ref. <50)** |  |  |  |  |  |  |
| 50-64 | 0.74 | 0.45, 1.21 | 0.229 | 0.72 | 0.43 – 1.20 | 0.212 |
| >65 | 0.34 | 0.20, 0.58 | **<0.001** | 0.34 | 0.19 – 0.61 | **<0.001** |
| **Education (Ref. Primary or middle**  **school)** |  |  |  |  |  |  |
| High school | 1.51 | 0.96, 2.39 | **0.077** |  |  |  |
| University degree or higher | 1.41 | 0.79, 2.56 | 0.252 |  |  |  |
| **Time since diagnosis, months (Ref. 1-**  **6)** |  |  |  |  |  |  |
| 7-12 | 0.94 | 0.57, 1.58 | 0.822 | 1.13 | 0.65 – 1.97 | 0.666 |
| 13-18 | 1.36 | 0.76, 2.48 | 0.304 | 1.65 | 0.88 – 3.10 | 0.117 |
| 19-24 | 1.66 | 0.92, 3.10 | **0.101** | 2.31 | 1.19 – 4.50 | **0.014** |
| **Menopausal status (Ref. Pre)** |  |  |  |  |  |  |
| Post | 0.60 | 0.39, 0.91 | **0.017** |  |  |  |
| **HER2 status after treatment (Ref.**  **Positive)** |  |  |  |  |  |  |
| Negative | 1.39 | 0.89, 2.19 | **0.147** |  |  |  |
| **Tumor histotype after treatment**  **(Ref. Invasive ductal carcinoma**  **(IDC))** |  |  |  |  |  |  |
| Invasive lobular carcinoma (ILC) | 0.56 | 0.26, 1.18 | **0.121** |  |  |  |
| Other | 0.68 | 0.32, 1.48 | 0.323 |  |  |  |
| **Types of breast cancer surgery (Ref.**  **Breast-conserving)** |  |  |  |  |  |  |
| Mastectomy | 1.46 | 0.95, 2.27 | **0.086** |  |  |  |
| **Types of adjuvant therapy (Ref.**  **Chemotherapy or trastuzumab or**  **other)** |  |  |  |  |  |  |
| Hormone therapy | 0.56 | 0.36, 0.85 | **0.006** | 0.60 | 0.36 – 0.98 | **0.040** |
| **Types of adjuvant therapy (Ref.**  **Trastuzumab or other)** |  |  |  |  |  |  |
| Chemotherapy and hormonal therapy | 1.46 | 0.91, 2.40 | **0.125** |  |  |  |
| **Types of adjuvant therapy (Ref.**  **Other)** |  |  |  |  |  |  |
| Chemotherapy and hormonal therapy  and trastuzumab | 1.47 | 0.82, 2.75 | 0.211 |  |  |  |
| **Types of adjuvant therapy (Ref.**  **Chemotherapy or hormonal therapy**  **or trastuzumab)** |  |  |  |  |  |  |
| Other | 1.10 | 0.64, 1.93 | 0.745 |  |  |  |
| **Post-operative radiotherapy (Ref.**  **No)** |  |  |  |  |  |  |
| Yes | 0.86 | 0.56, 1.31 | 0.496 |  |  |  |
| NOTE. N: number of respondents included in the regression.  *Model was adjusted for participating oncology center.  ABBREVIATIONS. OR: Odds Ratio, CI: Confidence Interval, BMI: Body Mass Index, HER2: human epidermal growth factor receptor 2. | | | | | | |

SUPPLEMENTARY TABLE 3. Factors associated with the change in poultry consumption.

|  | **Poultry** | | | | | |
| --- | --- | --- | --- | --- | --- | --- |
|  | **Univariate analysis (N=402)** | | | **Multivariate analysis* (N=402)** | | |
| **Characteristics** | **OR** | **95% CI** | **P-value** | **OR** | **95% CI** | **P-value** |
| **BMI (Ref. Underweight or Normal**  **weight)** |  |  |  |  |  |  |
| Overweight or Obesity | 1.96 | 1.30, 2.96 | **0.001** | 1.89 | 1.19 – 3.02 | **0.007** |
| **Age (Ref. <50)** |  |  |  |  |  |  |
| 50-64 | 0.76 | 0.48, 1.19 | 0.232 | 0.61 | 0.38 – 1.00 | 0.052 |
| >65 | 0.65 | 0.38, 1.10 | **0.107** | 0.26 | 0.14 – 0.51 | **<0.001** |
| **Education (Ref. Primary or**  **middle school)** |  |  |  |  |  |  |
| High school | 1.42 | 0.90, 2.23 | **0.131** |  |  |  |
| University degree or higher | 1.12 | 0.63, 1.99 | 0.695 |  |  |  |
| **Time since diagnosis, months (Ref.**  **1-6)** |  |  |  |  |  |  |
| 7-12 | 0.84 | 0.49, 1.41 | 0.502 | 1.60 | 0.90 – 2.85 | 0.112 |
| 13-18 | 1.64 | 0.94, 2.88 | **0.080** | 2.62 | 1.41 – 4.90 | **0.002** |
| 19-24 | 1.25 | 0.71, 2.19 | 0.431 | 2.56 | 1.34 – 4.89 | **0.004** |
| **Menopausal status (Ref. Pre)** |  |  |  |  |  |  |
| Post | 0.87 | 0.59, 1.30 | 0.504 |  |  |  |
| **HER2 status after treatment (Ref.**  **Positive)** |  |  |  |  |  |  |
| Negative | 1.23 | 0.80, 1.88 | 0.340 |  |  |  |
| **Tumor histotype after treatment**  **(Ref. Invasive ductal carcinoma**  **(IDC))** |  |  |  |  |  |  |
| Invasive lobular carcinoma (ILC) | 0.67 | 0.30, 1.42 | 0.305 |  |  |  |
| Other | 0.81 | 0.37, 1.74 | 0.600 |  |  |  |
| **Types of breast cancer surgery**  **(Ref. Breast-conserving)** |  |  |  |  |  |  |
| Mastectomy | 1.71 | 1.13, 2.59 | **0.011** |  |  |  |
| **Types of adjuvant therapy (Ref.**  **Chemotherapy or trastuzumab or**  **other)** |  |  |  |  |  |  |
| Hormone therapy | 0.58 | 0.38, 0.89 | **0.012** | 0.62 | 0.37 – 1.02 | 0.059 |
| **Types of adjuvant therapy (Ref.**  **Trastuzumab or other)** |  |  |  |  |  |  |
| Chemotherapy and hormonal therapy | 1.32 | 0.84, 2.08 | 0.229 |  |  |  |
| **Types of adjuvant therapy (Ref.**  **Other)** |  |  |  |  |  |  |
| Chemotherapy and hormonal therapy  and trastuzumab | 1.30 | 0.74, 2.26 | 0.358 |  |  |  |
| **Types of adjuvant therapy (Ref.**  **Chemotherapy or hormonal**  **therapy or trastuzumab)** |  |  |  |  |  |  |
| Other | 0.75 | 0.43, 1.27 | 0.289 |  |  |  |
| **Post-operative radiotherapy (Ref.**  **No)** |  |  |  |  |  |  |
| Yes | 0.74 | 0.49, 1.11 | **0.142** |  |  |  |
| NOTE. N: number of respondents included in the regression.  *Model was adjusted for participating oncology center.  ABBREVIATIONS. OR: Odds Ratio, CI: Confidence Interval, BMI: Body Mass Index, HER2: human epidermal growth factor receptor 2. | | | | | | |

SUPPLEMENTARY TABLE 4. Factors associated with the change in fruits, vegetables, pulses and nuts consumption.

|  | **Fruits, vegetables, pulses and nuts** | | | | | |
| --- | --- | --- | --- | --- | --- | --- |
|  | **Univariate analysis (N=399)** | | | **Multivariate analysis* (N=399)** | | |
| **Characteristics** | **OR** | **95% CI** | **P-value** | **OR** | **95% CI** | **P-value** |
| **BMI (Ref. Underweight or Normal**  **weight)** |  |  |  |  |  |  |
| Overweight or Obesity | 1.50 | 0.99, 2.31 | **0.059** | 1.77 | 1.11 – 2.83 | **0.017** |
| **Age (Ref. <50)** |  |  |  |  |  |  |
| 50-64 | 0.80 | 0.49, 1.28 | 0.353 | 0.78 | 0.47 – 1.29 | 0.337 |
| >65 | 0.39 | 0.23, 0.67 | **<0.001** | 0.39 | 0.21 – 0.72 | **0.002** |
| **Education (Ref. Primary or middle**  **school)** |  |  |  |  |  |  |
| High school | 1.33 | 0.84, 2.09 | 0.220 |  |  |  |
| University degree or higher | 1.69 | 0.93, 3.09 | **0.086** |  |  |  |
| **Time since diagnosis, months (Ref.**  **1-6)** |  |  |  |  |  |  |
| 7-12 | 1.09 | 0.65, 1.84 | 0.736 |  |  |  |
| 13-18 | 1.25 | 0.70, 2.25 | 0.456 |  |  |  |
| 19-24 | 1.08 | 0.61, 1.93 | 0.791 |  |  |  |
| **Menopausal status (Ref. Pre)** |  |  |  |  |  |  |
| Post | 0.72 | 0.48, 1.09 | **0.124** |  |  |  |
| **HER2 status after treatment (Ref.**  **Positive)** |  |  |  |  |  |  |
| Negative | 1.15 | 0.74, 1.78 | 0.542 |  |  |  |
| **Tumor histotype after treatment**  **(Ref. Invasive ductal carcinoma**  **(IDC))** |  |  |  |  |  |  |
| Invasive lobular carcinoma (ILC) | 0.60 | 0.29, 1.27 | **0.179** |  |  |  |
| Other | 0.56 | 0.27, 1.20 | **0.135** |  |  |  |
| **Types of breast cancer surgery (Ref.**  **Breast-conserving)** |  |  |  |  |  |  |
| Mastectomy | 1.18 | 0.78, 1.81 | 0.439 |  |  |  |
| **Types of adjuvant therapy (Ref.**  **Chemotherapy or trastuzumab or**  **other)** |  |  |  |  |  |  |
| Hormone therapy | 0.47 | 0.31, 0.71 | **<0.001** | 0.56 | 0.34 – 0.90 | **0.017** |
| **Types of adjuvant therapy (Ref.**  **Trastuzumab or other)** |  |  |  |  |  |  |
| Chemotherapy and hormonal therapy | 1.67 | 1.04, 2.75 | **0.037** |  |  |  |
| **Types of adjuvant therapy (Ref.**  **Other)** |  |  |  |  |  |  |
| Chemotherapy and hormonal therapy  and Trastuzumab | 1.07 | 0.61, 1.92 | 0.805 |  |  |  |
| **Types of adjuvant therapy (Ref.**  **Chemotherapy or hormonal**  **therapy or trastuzumab)** |  |  |  |  |  |  |
| Other | 1.25 | 0.72, 2.21 | 0.441 |  |  |  |
| **Post-operative radiotherapy (Ref.**  **No)** |  |  |  |  |  |  |
| Yes | 1.02 | 0.67, 1.54 | 0.944 |  |  |  |
| NOTE. N: number of respondents included in the regression.  *Model was adjusted for participating oncology center.  ABBREVIATIONS. OR: Odds Ratio, CI: Confidence Interval, BMI: Body Mass Index, HER2: human epidermal growth factor receptor 2. | | | | | | |

SUPPLEMENTARY TABLE 5. Factors associated with the change in milk and dairy products consumption.

|  | **Milk and dairy products** | | | | | |
| --- | --- | --- | --- | --- | --- | --- |
|  | **Univariate analysis (N=391)** | | | **Multivariate analysis* (N=391)** | | |
| **Characteristics** | **OR** | **95% CI** | **P-value** | **OR** | **95% CI** | **P-value** |
| **BMI (Ref. Underweight or Normal**  **weight)** |  |  |  |  |  |  |
| Overweight or Obesity | 0.94 | 0.61, 1.45 | 0.797 |  |  |  |
| **Age (Ref. <50)** |  |  |  |  |  |  |
| 50-64 | 0.72 | 0.45, 1.14 | **0.161** | 0.61 | 0.37 – 1.02 | 0.058 |
| >65 | 0.47 | 0.26, 0.82 | **0.010** | 0.38 | 0.20 – 0.73 | **0.004** |
| **Education (Ref. Primary or middle**  **school)** |  |  |  |  |  |  |
| High school | 1.64 | 1.02, 2.65 | **0.041** | 1.49 | 0.91 – 2.44 | 0.116 |
| University degree or higher | 0.85 | 0.44, 1.58 | 0.604 | 0.52 | 0.26 – 1.05 | 0.067 |
| **Time since diagnosis, months (Ref.**  **1-6)** |  |  |  |  |  |  |
| 7-12 | 1.16 | 0.67, 2.01 | 0.592 |  |  |  |
| 13-18 | 1.76 | 0.99, 3.12 | **0.054** |  |  |  |
| 19-24 | 1.38 | 0.76, 2.48 | 0.282 |  |  |  |
| **Menopausal status (Ref. Pre)** |  |  |  |  |  |  |
| Post | 0.68 | 0.45, 1.03 | **0.070** |  |  |  |
| **HER2 status after treatment (Ref.**  **Positive)** |  |  |  |  |  |  |
| Negative | 0.98 | 0.62, 1.52 | 0.915 |  |  |  |
| **Tumor histotype after treatment**  **(Ref. Invasive ductal carcinoma**  **(IDC))** |  |  |  |  |  |  |
| Invasive lobular carcinoma (ILC) | 0.41 | 0.15, 0.96 | **0.055** |  |  |  |
| Other | 1.35 | 0.62, 2.86 | 0.437 |  |  |  |
| **Types of breast cancer surgery (Ref.**  **Breast-conserving)** |  |  |  |  |  |  |
| Mastectomy | 1.17 | 0.76, 1.79 | 0.476 |  |  |  |
| **Types of adjuvant therapy (Ref.**  **Chemotherapy or trastuzumab or**  **other)** |  |  |  |  |  |  |
| Hormone therapy | 0.67 | 0.42, 1.04 | **0.076** | 0.64 | 0.38 – 1.06 | 0.080 |
| **Types of adjuvant therapy (Ref.**  **Trastuzumab or other)** |  |  |  |  |  |  |
| Chemotherapy and hormonal therapy | 1.27 | 0.79, 2.02 | 0.324 |  |  |  |
| **Types of adjuvant therapy (Ref.**  **Other)** |  |  |  |  |  |  |
| Chemotherapy and hormonal therapy  and Trastuzumab | 1.11 | 0.62, 1.95 | 0.728 |  |  |  |
| **Types of adjuvant therapy (Ref.**  **Chemotherapy or hormonal**  **therapy or trastuzumab)** |  |  |  |  |  |  |
| Other | 0.76 | 0.42, 1.33 | 0.353 |  |  |  |
| **Post-operative radiotherapy (Ref.**  **No)** |  |  |  |  |  |  |
| Yes | 0.95 | 0.62, 1.46 | 0.818 |  |  |  |
| NOTE. N: number of respondents included in the regression.  *Model was adjusted for participating oncology center.  ABBREVIATIONS. OR: Odds Ratio, CI: Confidence Interval, BMI: Body Mass Index, HER2: human epidermal growth factor receptor 2. | | | | | | |

SUPPLEMENTARY TABLE 6. Factors associated with the change in eggs consumption.

|  | **Eggs** | | | | | |
| --- | --- | --- | --- | --- | --- | --- |
|  | **Univariate analysis (N=404)** | | | **Multivariate analysis* (N=404)** | | |
| **Characteristics** | **OR** | **95% CI** | **P-value** | **OR** | **95% CI** | **P-value** |
| **BMI (Ref. Underweight or**  **Normal weight)** |  |  |  |  |  |  |
| Overweight or Obesity | 1.45 | 0.89, 2.36 | **0.130** | 1.97 | 1.15 – 3.39 | **0.014** |
| **Age (Ref. <50)** |  |  |  |  |  |  |
| 50-64 | 1.22 | 0.73, 2.06 | 0.458 | 1.15 | 0.66 – 1.99 | 0.620 |
| >65 | 0.25 | 0.10, 0.57 | **0.002** | 0.18 | 0.07 – 0.47 | **<0.001** |
| **Education (Ref. Primary or**  **middle school)** |  |  |  |  |  |  |
| High school | 0.83 | 0.48, 1.43 | 0.494 |  |  |  |
| University degree or higher | 1.18 | 0.60, 2.29 | 0.618 |  |  |  |
| **Time since diagnosis, months**  **(Ref. 1-6)** |  |  |  |  |  |  |
| 7-12 | 0.89 | 0.46, 1.70 | 0.730 | 0.81 | 0.40 – 1.63 | 0.551 |
| 13-18 | 2.27 | 1.22, 4.22 | **0.009** | 2.13 | 1.09 – 4.14 | **0.027** |
| 19-24 | 0.76 | 0.34, 1.57 | 0.466 | 0.66 | 0.30 – 1.48 | 0.312 |
| **Menopausal status (Ref. Pre)** |  |  |  |  |  |  |
| Post | 0.61 | 0.37, 0.98 | **0.041** |  |  |  |
| **HER2 status after treatment**  **(Ref. Positive)** |  |  |  |  |  |  |
| Negative | 0.84 | 0.49, 1.40 | 0.504 |  |  |  |
| **Tumor histotype after**  **treatment (Ref. Invasive**  **ductal carcinoma (IDC))** |  |  |  |  |  |  |
| Invasive lobular carcinoma  (ILC) | 1.07 | 0.41, 2.45 | 0.882 |  |  |  |
| Other | 1.16 | 0.45, 2.69 | 0.740 |  |  |  |
| **Types of breast cancer surgery**  **(Ref. Breast-conserving)** |  |  |  |  |  |  |
| Mastectomy | 1.10 | 0.67, 1.80 | 0.704 |  |  |  |
| **Types of adjuvant therapy**  **(Ref. Chemotherapy or**  **trastuzumab or other)** |  |  |  |  |  |  |
| Hormone therapy | 0.61 | 0.35, 1.02 | **0.065** |  |  |  |
| **Types of adjuvant therapy**  **(Ref. Trastuzumab or other)** |  |  |  |  |  |  |
| Chemotherapy and hormonal  therapy | 1.63 | 0.96, 2.72 | **0.068** |  |  |  |
| **Types of adjuvant therapy**  **(Ref. Other)** |  |  |  |  |  |  |
| Chemotherapy and hormonal  therapy and trastuzumab | 1.02 | 0.51, 1.93 | 0.955 |  |  |  |
| **Types of adjuvant therapy**  **(Ref. Chemotherapy or**  **hormonal therapy or**  **trastuzumab)** |  |  |  |  |  |  |
| Other | 1.35 | 0.71, 2.44 | 0.342 |  |  |  |
| **Post-operative radiotherapy**  **(Ref. No)** |  |  |  |  |  |  |
| Yes | 1.35 | 0.82, 2.25 | 0.248 |  |  |  |
| NOTE. N: number of respondents included in the regression.  *Model was adjusted for participating oncology center.  ABBREVIATIONS. OR: Odds Ratio, CI: Confidence Interval, BMI: Body Mass Index, HER2: human epidermal growth factor receptor 2. | | | | | | |

SUPPLEMENTARY TABLE 7. Factors associated with the change of baked goods, refined bread and pasta consumption.

|  | **Baked goods and refined bread and pasta** | | | | | |
| --- | --- | --- | --- | --- | --- | --- |
|  | **Univariate analysis (N=393)** | | | **Multivariate analysis* (N=393)** | | |
| **Characteristics** | **OR** | **95% CI** | **P-value** | **OR** | **95% CI** | **P-value** |
| **BMI (Ref. Underweight or**  **Normal weight)** |  |  |  |  |  |  |
| Overweight or Obesity | 1.58 | 1.04, 2.44 | **0.035** | 2.20 | 1.33 – 3.64 | **0.002** |
| **Age (Ref. <50)** |  |  |  |  |  |  |
| 50-64 | 0.72 | 0.44, 1.17 | **0.184** | 0.50 | 0.25 – 0.97 | **0.041** |
| >65 | 0.34 | 0.19, 0.59 | **<0.001** | 0.17 | 0.07 – 0.42 | **<0.001** |
| **Education (Ref. Primary or**  **middle school)** |  |  |  |  |  |  |
| High school | 1.55 | 0.98, 2.44 | **0.061** | 1.42 | 0.86 – 2.34 | 0.173 |
| University degree or higher | 1.99 | 1.10, 3.68 | **0.024** | 2.14 | 1.06 – 4.32 | **0.033** |
| **Time since diagnosis, months**  **(Ref. 1-6)** |  |  |  |  |  |  |
| 7-12 | 1.56 | 0.92, 2.65 | **0.099** | 1.70 | 0.94 – 3.05 | 0.077 |
| 13-18 | 1.85 | 1.04, 3.37 | **0.04** | 2.17 | 1.12 – 4.17 | **0.021** |
| 19-24 | 1.81 | 1.01, 3.30 | **0.048** | 2.26 | 1.16 – 4.42 | **0.017** |
| **Menopausal status (Ref. Pre)** |  |  |  |  |  |  |
| Post | 0.75 | 0.49, 1.12 | **0.164** | 1.79 | 0.88 – 3.67 | 0.110 |
| **HER2 status after treatment**  **(Ref. Positive)** |  |  |  |  |  |  |
| Negative | 1.23 | 0.79, 1.91 | 0.362 |  |  |  |
| **Tumor histotype after**  **treatment (Ref. Invasive**  **ductal carcinoma (IDC))** |  |  |  |  |  |  |
| Invasive lobular carcinoma  (ILC) | 0.59 | 0.28, 1.26 | **0.172** |  |  |  |
| Other | 0.78 | 0.37, 1.68 | 0.510 |  |  |  |
| **Types of breast cancer surgery**  **(Ref. Breast-conserving)** |  |  |  |  |  |  |
| Mastectomy | 1.71 | 1.11, 2.66 | **0.015** | 1.77 | 1.08 – 2.90 | **0.023** |
| **Types of adjuvant therapy**  **(Ref. Chemotherapy or**  **trastuzumab or other)** |  |  |  |  |  |  |
| Hormone therapy | 0.56 | 0.37, 0.86 | **0.007** |  |  |  |
| **Types of adjuvant therapy**  **(Ref. Trastuzumab or other)** |  |  |  |  |  |  |
| Chemotherapy and hormonal  therapy | 1.41 | 0.88, 2.31 | **0.159** |  |  |  |
| **Types of adjuvant therapy**  **(Ref. Other)** |  |  |  |  |  |  |
| Chemotherapy and hormonal  therapy and trastuzumab | 1.17 | 0.66, 2.11 | 0.598 |  |  |  |
| **Types of adjuvant therapy**  **(Ref. Chemotherapy or**  **hormonal therapy or**  **trastuzumab)** |  |  |  |  |  |  |
| Other | 1.15 | 0.66, 2.03 | 0.623 |  |  |  |
| **Post-operative radiotherapy**  **(Ref. No)** |  |  |  |  |  |  |
| Yes | 0.88 | 0.58, 1.34 | 0.553 |  |  |  |
| NOTE. N: number of respondents included in the regression.  *Model was adjusted for participating oncology center.  ABBREVIATIONS. OR: Odds Ratio, CI: Confidence Interval, BMI: Body Mass Index, HER2: human epidermal growth factor receptor 2. | | | | | | |

SUPPLEMENTARY TABLE 8. Factors associated with the change in fish and shellfish consumption.

|  | **Fish and shellfish** | | | | | |
| --- | --- | --- | --- | --- | --- | --- |
|  | **Univariate analysis (N=401)** | | | **Multivariate analysis* (N=401)** | | |
| **Characteristics** | **OR** | **95% CI** | **P-value** | **OR** | **95% CI** | **P-value** |
| **BMI (Ref. Underweight or**  **Normal weight)** |  |  |  |  |  |  |
| Overweight or Obesity | 1.32 | 0.86, 2.01 | **0.198** | 1.55 | 0.98 – 2.46 | **0.062** |
| **Age (Ref. <50)** |  |  |  |  |  |  |
| 50-64 | 0.71 | 0.45, 1.13 | **0.151** | 0.66 | 0.40 – 1.08 | 0.096 |
| >65 | 0.49 | 0.28, 0.86 | **0.014** | 0.43 | 0.23 – 0.82 | **0.010** |
| **Education (Ref. Primary or**  **middle school)** |  |  |  |  |  |  |
| High school | 0.99 | 0.62, 1.59 | 0.962 |  |  |  |
| University degree or higher | 1.47 | 0.82, 2.64 | **0.195** |  |  |  |
| **Time since diagnosis, months**  **(Ref. 1-6)** |  |  |  |  |  |  |
| 7-12 | 0.79 | 0.46, 1.35 | 0.396 |  |  |  |
| 13-18 | 1.09 | 0.61, 1.94 | 0.763 |  |  |  |
| 19-24 | 1.03 | 0.57, 1.83 | 0.922 |  |  |  |
| **Menopausal status (Ref. Pre)** |  |  |  |  |  |  |
| Post | 0.70 | 0.46, 1.06 | **0.092** |  |  |  |
| **HER2 status after treatment**  **(Ref. Positive)** |  |  |  |  |  |  |
| Negative | 1.09 | 0.70, 1.68 | 0.714 |  |  |  |
| **Tumor histotype after**  **treatment (Ref. Invasive ductal**  **carcinoma (IDC))** |  |  |  |  |  |  |
| Invasive lobular carcinoma (ILC) | 0.91 | 0.40, 1.95 | 0.809 |  |  |  |
| Other | 1.27 | 0.58, 2.70 | 0.539 |  |  |  |
| **Types of breast cancer surgery**  **(Ref. Breast-conserving)** |  |  |  |  |  |  |
| Mastectomy | 1.49 | 0.97, 2.28 | **0.065** | 1.48 | 0.93 – 2.36 | 0.096 |
| **Types of adjuvant therapy**  **(Ref. Chemotherapy or**  **trastuzumab or other)** |  |  |  |  |  |  |
| Hormone therapy | 0.61 | 0.39, 0.95 | **0.031** | 0.67 | 0.41 – 1.11 | 0.120 |
| **Types of adjuvant therapy**  **(Ref. Trastuzumab or other)** |  |  |  |  |  |  |
| Chemotherapy and hormonal  therapy | 1.26 | 0.79, 2.00 | 0.335 |  |  |  |
| **Types of adjuvant therapy**  **(Ref. Other)** |  |  |  |  |  |  |
| Chemotherapy and hormonal  therapy and trastuzumab | 0.88 | 0.48, 1.57 | 0.667 |  |  |  |
| **Types of adjuvant therapy**  **(Ref. Chemotherapy or**  **hormonal therapy or**  **trastuzumab)** |  |  |  |  |  |  |
| Other | 1.44 | 0.84, 2.46 | **0.181** |  |  |  |
| **Post-operative radiotherapy**  **(Ref. No)** |  |  |  |  |  |  |
| Yes | 1.03 | 0.68, 1.58 | 0.882 |  |  |  |
| NOTE. N: number of respondents included in the regression.  *Model was adjusted for participating oncology center.  ABBREVIATIONS. OR: Odds Ratio, CI: Confidence Interval, BMI: Body Mass Index, HER2: human epidermal growth factor receptor 2. | | | | | | |

SUPPLEMENTARY TABLE 9. Factors associated with the change in hand-made sweet products and soft drinks consumption.

|  | **Hand-made sweet products and soft drinks** | | | | | |
| --- | --- | --- | --- | --- | --- | --- |
|  | **Univariate analysis (N=396)** | | | **Multivariate analysis* (N=396)** | | |
| **Characteristics** | **OR** | **95% CI** | **P-value** | **OR** | **95% CI** | **P-value** |
| **BMI (Ref. Underweight or**  **Normal weight)** |  |  |  |  |  |  |
| Overweight or Obesity | 1.31 | 0.86, 2.03 | 0.217 |  |  |  |
| **Age (Ref. <50)** |  |  |  |  |  |  |
| 50-64 | 0.91 | 0.55, 1.49 | 0.715 | 0.97 | 0.57 – 1.65 | 0.904 |
| >65 | 0.32 | 0.18, 0.56 | **<0.001** | 0.26 | 0.14 – 0.49 | **<0.001** |
| **Education (Ref. Primary or**  **middle school)** |  |  |  |  |  |  |
| High school | 1.47 | 0.92, 2.33 | **0.106** |  |  |  |
| University degree or higher | 1.49 | 0.83, 2.72 | **0.188** |  |  |  |
| **Time since diagnosis, months**  **(Ref. 1-6)** |  |  |  |  |  |  |
| 7-12 | 1.21 | 0.72, 2.05 | 0.472 | 1.32 | 0.73 – 2.40 | 0.363 |
| 13-18 | 2.18 | 1.19, 4.14 | **0.014** | 2.65 | 1.33 – 5.27 | **0.005** |
| 19-24 | 1.56 | 0.87, 2.87 | **0.145** | 2.08 | 1.05 – 4.10 | **0.036** |
| **Menopausal status (Ref. Pre)** |  |  |  |  |  |  |
| Post | 0.79 | 0.52, 1.19 | 0.257 |  |  |  |
| **HER2 status after treatment**  **(Ref. Positive)** |  |  |  |  |  |  |
| Negative | 1.40 | 0.90, 2.21 | **0.141** |  |  |  |
| **Tumor histotype after treatment**  **(Ref. Invasive ductal carcinoma**  **(IDC))** |  |  |  |  |  |  |
| Invasive lobular carcinoma (ILC) | 0.80 | 0.38, 1.76 | 0.570 |  |  |  |
| Other | 0.80 | 0.38, 1.76 | 0.570 |  |  |  |
| **Types of breast cancer surgery**  **(Ref. Breast-conserving)** |  |  |  |  |  |  |
| Mastectomy | 2.14 | 1.37, 3.40 | **<0.001** | 2.12 | 1.28 – 3.52 | **0.004** |
| **Types of adjuvant therapy (Ref.**  **Chemotherapy or trastuzumab**  **or other)** |  |  |  |  |  |  |
| Hormone therapy | 0.53 | 0.34, 0.81 | **0.003** | 0.61 | 0.36 – 1.03 | 0.064 |
| **Types of adjuvant therapy (Ref.**  **Trastuzumab or other)** |  |  |  |  |  |  |
| Chemotherapy and hormonal  therapy | 1.32 | 0.82, 2.16 | 0.267 |  |  |  |
| **Types of adjuvant therapy (Ref.**  **Other)** |  |  |  |  |  |  |
| Chemotherapy and hormonal  therapy and trastuzumab | 1.31 | 0.73, 2.43 | 0.376 |  |  |  |
| **Types of adjuvant therapy (Ref.**  **Chemotherapy or hormonal**  **therapy or trastuzumab)** |  |  |  |  |  |  |
| Other | 1.63 | 0.92, 2.98 | **0.103** |  |  |  |
| **Post-operative radiotherapy (Ref.**  **No)** |  |  |  |  |  |  |
| Yes | 0.84 | 0.54, 1.28 | 0.416 |  |  |  |
| NOTE. N: number of respondents included in the regression.  *Model was adjusted for participating oncology center.  ABBREVIATIONS. OR: Odds Ratio, CI: Confidence Interval, BMI: Body Mass Index, HER2: human epidermal growth factor receptor 2. | | | | | | |

SUPPLEMENTARY TABLE 10. Factors associated with the change in wholemeal bread or pasta and grain consumption.

|  | **Wholemeal bread or pasta and grains** | | | | | |
| --- | --- | --- | --- | --- | --- | --- |
|  | **Univariate analysis (N=369)** | | | **Multivariate analysis* (N=069)** | | |
| **Characteristics** | **OR** | **95% CI** | **P-value** | **OR** | **95% CI** | **P-value** |
| **BMI (Ref. Underweight or**  **Normal weight)** |  |  |  |  |  |  |
| Overweight or Obesity | 1.73 | 1.13, 2.67 | **0.012** | 2.45 | 1.47 – 4.09 | **0.001** |
| **Age (Ref. <50)** |  |  |  |  |  |  |
| 50-64 | 0.90 | 0.56, 1.43 | 0.651 | 0.75 | 0.45 – 1.25 | 0.264 |
| >65 | 0.40 | 0.22, 0.70 | **0.002** | 0.27 | 0.13 – 0.53 | **<0.001** |
| **Education (Ref. Primary or**  **middle school)** |  |  |  |  |  |  |
| High school | 1.31 | 0.82, 2.09 | 0.259 |  |  |  |
| University degree or higher | 1.37 | 0.76, 2.48 | 0.291 |  |  |  |
| **Time since diagnosis, months**  **(Ref. 1-6)** |  |  |  |  |  |  |
| 7-12 | 1.69 | 0.99, 2.89 | **0.054** | 1.76 | 0.97 – 3.17 | 0.061 |
| 13-18 | 1.57 | 0.89, 2.80 | **0.122** | 1.53 | 0.82 – 2.87 | 0.181 |
| 19-24 | 2.58 | 1.42, 4.81 | **0.002** | 2.93 | 1.47 – 5.83 | **0.002** |
| **Menopausal status (Ref. Pre)** |  |  |  |  |  |  |
| Post | 0.85 | 0.56, 1.28 | 0.439 |  |  |  |
| **HER2 status after treatment**  **(Ref. Positive)** |  |  |  |  |  |  |
| Negative | 1.02 | 0.66, 1.58 | 0.942 |  |  |  |
| **Tumor histotype after**  **treatment (Ref. Invasive ductal**  **carcinoma (IDC))** |  |  |  |  |  |  |
| Invasive lobular carcinoma  (ILC) | 0.86 | 0.38, 1.94 | 0.72 |  |  |  |
| Other | 0.86 | 0.40, 1.89 | 0.711 |  |  |  |
| **Types of breast cancer surgery**  **(Ref. Breast-conserving)** |  |  |  |  |  |  |
| Mastectomy | 1.31 | 0.86, 2.01 | 0.213 |  |  |  |
| **Types of adjuvant therapy**  **(Ref. Chemotherapy or**  **trastuzumab or other)** |  |  |  |  |  |  |
| Hormone therapy | 0.52 | 0.34, 0.80 | **0.003** | 0.64 | 0.38 – 1.07 | 0.090 |
| **Types of adjuvant therapy**  **(Ref. Trastuzumab or other)** |  |  |  |  |  |  |
| Chemotherapy and hormonal  therapy | 1.56 | 0.97, 2.54 | **0.069** |  |  |  |
| **Types of adjuvant therapy**  **(Ref. Other)** |  |  |  |  |  |  |
| Chemotherapy and hormonal  therapy and trastuzumab | 1.13 | 0.63, 2.06 | 0.679 |  |  |  |
| **Types of adjuvant therapy**  **(Ref. Chemotherapy or**  **hormonal therapy or**  **trastuzumab)** |  |  |  |  |  |  |
| Other | 1.13 | 0.65, 1.98 | 0.654 |  |  |  |
| **Post-operative radiotherapy**  **(Ref. No)** |  |  |  |  |  |  |
| Yes | 1.15 | 0.75, 1.74 | 0.527 |  |  |  |
| NOTE. N: number of respondents included in the regression.  *Model was adjusted for participating oncology center.  ABBREVIATIONS. OR: Odds Ratio, CI: Confidence Interval, BMI: Body Mass Index, HER2: human epidermal growth factor receptor 2. | | | | | | |

SUPPLEMENTARY TABLE 11. Factors associated with the change in preserved fish consumption.

|  | **Preserved fish** | | | | | |
| --- | --- | --- | --- | --- | --- | --- |
|  | **Univariate analysis (N=397)** | | | **Multivariate analysis* (N=397)** | | |
| **Characteristics** | **OR** | **95% CI** | **P-value** | **OR** | **95% CI** | **P-value** |
| **BMI (Ref. Underweight or Normal**  **weight)** |  |  |  |  |  |  |
| Overweight or Obesity | 1.51 | 0.96, 2.37 | **0.071** | 1.83 | 1.10 – 3.04 | 0.019 |
| **Age (Ref. <50)** |  |  |  |  |  |  |
| 50-64 | 0.81 | 0.49, 1.32 | 0.389 | 0.63 | 0.36 – 1.09 | 0.099 |
| >65 | 0.53 | 0.28, 0.98 | **0.046** | 0.36 | 0.17 – 0.75 | **0.007** |
| **Education (Ref. Primary or middle**  **school)** |  |  |  |  |  |  |
| High school | 1.75 | 1.06, 2.95 | **0.031** | 1.56 | 0.90 – 2.70 | 0.110 |
| University degree or higher | 0.86 | 0.42, 1.72 | 0.678 | 0.58 | 0.26 – 1.26 | 0.165 |
| **Time since diagnosis, months (Ref.**  **1-6)** |  |  |  |  |  |  |
| 7-12 | 1.50 | 0.85, 2.64 | **0.162** |  |  |  |
| 13-18 | 1.38 | 0.73, 2.58 | 0.310 |  |  |  |
| 19-24 | 1.38 | 0.73, 2.58 | 0.310 |  |  |  |
| **Menopausal status (Ref. Pre)** |  |  |  |  |  |  |
| Post | 0.87 | 0.56, 1.36 | 0.545 |  |  |  |
| **HER2 status after treatment (Ref.**  **Positive)** |  |  |  |  |  |  |
| Negative | 1.52 | 0.96, 2.41 | **0.075** | 2.04 | 1.16 – 3.59 | **0.013** |
| **Tumor histotype after treatment**  **(Ref. Invasive ductal carcinoma**  **(IDC))** |  |  |  |  |  |  |
| Invasive lobular carcinoma (ILC) | 0.50 | 0.16, 1.23 | **0.162** |  |  |  |
| Other | 1.29 | 0.56, 2.79 | 0.534 |  |  |  |
| **Types of breast cancer surgery (Ref.**  **Breast-conserving)** |  |  |  |  |  |  |
| Mastectomy | 1.16 | 0.73, 1.83 | 0.521 |  |  |  |
| **Types of adjuvant therapy (Ref.**  **Chemotherapy or trastuzumab or**  **other)** |  |  |  |  |  |  |
| Hormone therapy | 0.43 | 0.25, 0.70 | **0.001** |  |  |  |
| **Types of adjuvant therapy (Ref.**  **Trastuzumab or other)** |  |  |  |  |  |  |
| Chemotherapy and hormonal therapy | 1.66 | 1.01, 2.69 | **0.043** | 2.01 | 1.10 – 3.66 | **0.022** |
| **Types of adjuvant therapy (Ref.**  **Other)** |  |  |  |  |  |  |
| Chemotherapy and hormonal therapy  and trastuzumab | 1.52 | 0.83, 2.74 | **0.165** |  |  |  |
| **Types of adjuvant therapy (Ref.**  **Chemotherapy or hormonal**  **therapy or trastuzumab)** |  |  |  |  |  |  |
| Other | 1.15 | 0.64, 2.03 | 0.630 |  |  |  |
| **Post-operative radiotherapy (Ref.**  **No)** |  |  |  |  |  |  |
| Yes | 1.16 | 0.74, 1.84 | 0.527 |  |  |  |
| NOTE. N: number of respondents included in the regression.  *Model was adjusted for participating oncology center.  ABBREVIATIONS. OR: Odds Ratio, CI: Confidence Interval, BMI: Body Mass Index, HER2: human epidermal growth factor receptor 2. | | | | | | |

SUPPLEMENTARY TABLE 12. Factors associated with the change in animal fats consumption.

|  | **Animal fats** | | | | | |
| --- | --- | --- | --- | --- | --- | --- |
|  | **Univariate analysis (N=397)** | | | **Multivariate analysis* (N=397)** | | |
| **Characteristics** | **OR** | **95% CI** | **P-value** | **OR** | **95% CI** | **P-value** |
| **BMI (Ref. Underweight or Normal**  **weight)** |  |  |  |  |  |  |
| Overweight or Obesity | 1.96 | 1.30, 2.96 | **0.001** | 2.56 | 1.62 – 4.05 | **<0.001** |
| **Age (Ref. <50)** |  |  |  |  |  |  |
| 50-64 | 0.76 | 0.48, 1.19 | 0.232 | 0.71 | 0.44 – 1.16 | 0.169 |
| >65 | 0.65 | 0.38, 1.10 | **0.107** | 0.47 | 0.26 – 0.86 | **0.015** |
| **Education (Ref. Primary or**  **Middle school)** |  |  |  |  |  |  |
| High school | 1.42 | 0.90, 2.23 | **0.131** |  |  |  |
| University degree or higher | 1.12 | 0.63, 1.99 | 0.695 |  |  |  |
| **Time since diagnosis, months (Ref.**  **1-6)** |  |  |  |  |  |  |
| 7-12 | 0.84 | 0.49, 1.41 | 0.502 |  |  |  |
| 13-18 | 1.64 | 0.94, 2.88 | **0.080** |  |  |  |
| 19-24 | 1.25 | 0.71, 2.19 | 0.431 |  |  |  |
| **Menopausal status (Ref. Pre)** |  |  |  |  |  |  |
| Post | 0.87 | 0.59, 1.30 | 0.504 |  |  |  |
| **HER2 status after treatment (Ref.**  **Positive)** |  |  |  |  |  |  |
| Negative | 1.23 | 0.80, 1.88 | 0.340 |  |  |  |
| **Tumor histotype after treatment**  **(Ref. Invasive ductal carcinoma**  **(IDC))** |  |  |  |  |  |  |
| Invasive lobular carcinoma (ILC) | 0.67 | 0.30, 1.42 | 0.305 |  |  |  |
| Other | 0.81 | 0.37, 1.74 | 0.600 |  |  |  |
| **Types of breast cancer surgery**  **(Ref. Breast-conserving)** |  |  |  |  |  |  |
| Mastectomy | 1.71 | 1.13, 2.59 | **0.011** | 1.71 | 1.09 – 2.68 | **0.021** |
| **Types of adjuvant therapy (Ref.**  **Chemotherapy or trastuzumab or**  **other)** |  |  |  |  |  |  |
| Hormone therapy | 0.58 | 0.38, 0.89 | **0.012** |  |  |  |
| **Types of adjuvant therapy (Ref.**  **Trastuzumab or other)** |  |  |  |  |  |  |
| Chemotherapy and hormonal therapy | 1.32 | 0.84, 2.08 | 0.229 |  |  |  |
| **Types of adjuvant therapy (Ref.**  **Other)** |  |  |  |  |  |  |
| Chemotherapy and hormonal therapy  and trastuzumab | 1.30 | 0.74, 2.26 | 0.358 |  |  |  |
| **Types of adjuvant therapy (Ref.**  **Chemotherapy or hormonal**  **therapy or trastuzumab)** |  |  |  |  |  |  |
| Other | 0.75 | 0.43, 1.27 | 0.289 |  |  |  |
| **Post-operative radiotherapy (Ref.**  **No)** |  |  |  |  |  |  |
| Yes | 0.74 | 0.49, 1.11 | **0.142** |  |  |  |
| NOTE. N: number of respondents included in the regression.  *Model was adjusted for participating oncology center.  ABBREVIATIONS. OR: Odds Ratio, CI: Confidence Interval, BMI: Body Mass Index, HER2: human epidermal growth factor receptor 2. | | | | | | |

SUPPLEMENTARY TABLE 13. Factors associated with the change in alcoholic drinks consumption.

|  | **Alcoholic drinks** | | | | | |
| --- | --- | --- | --- | --- | --- | --- |
|  | **Univariate analysis (N=398)** | | | **Multivariate analysis* (N=398)** | | |
| **Characteristics** | **OR** | **95% CI** | **P-value** | **OR** | **95% CI** | **P-value** |
| **BMI (Ref. Underweight or Normal**  **weight)** |  |  |  |  |  |  |
| Overweight or Obesity | 1.08 | 0.71, 1.66 | 0.709 |  |  |  |
| **Age (Ref. <50)** |  |  |  |  |  |  |
| 50-64 | 0.78 | 0.49, 1.25 | 0.304 |  |  |  |
| >65 | 0.47 | 0.26, 0.82 | **0.009** |  |  |  |
| **Education (Ref. Primary or middle**  **school)** |  |  |  |  |  |  |
| High school | 1.26 | 0.78, 2.08 | 0.350 | 1.07 | 0.64 – 1.80 | 0.798 |
| University degree or higher | 2.70 | 1.50, 4.91 | **0.001** | 1.89 | 0.98 – 3.65 | 0.059 |
| **Time since diagnosis, months (Ref.**  **1-6)** |  |  |  |  |  |  |
| 7-12 | 0.83 | 0.49, 1.41 | 0.498 |  |  |  |
| 13-18 | 0.93 | 0.52, 1.65 | 0.814 |  |  |  |
| 19-24 | 0.46 | 0.24, 0.86 | **0.019** |  |  |  |
| **Menopausal status (Ref. Pre)** |  |  |  |  |  |  |
| Post | 0.57 | 0.38, 0.87 | **0.009** | 0.58 | 0.35 – 0.96 | **0.035** |
| **HER2 status after treatment (Ref.**  **Positive)** |  |  |  |  |  |  |
| Negative | 2.32 | 1.50, 3.60 | **<0.001** | 1.70 | 1.02 – 2.86 | **0.044** |
| **Tumor histotype after treatment**  **(Ref. Invasive ductal carcinoma**  **(IDC))** |  |  |  |  |  |  |
| Invasive lobular carcinoma (ILC) | 0.63 | 0.26, 1.41 | 0.286 |  |  |  |
| Other | 0.86 | 0.36, 1.92 | 0.729 |  |  |  |
| **Types of breast cancer surgery (Ref.**  **Breast-conserving)** |  |  |  |  |  |  |
| Mastectomy | 1.49 | 0.97, 2.29 | **0.065** |  |  |  |
| **Types of adjuvant therapy (Ref.**  **Chemotherapy or trastuzumab or**  **other)** |  |  |  |  |  |  |
| Hormone therapy | 0.36 | 0.22, 0.57 | **<0.001** | 0.56 | 0.32 – 0.99 | **0.048** |
| **Types of adjuvant therapy (Ref.**  **Trastuzumab or other)** |  |  |  |  |  |  |
| Chemotherapy and hormonal therapy | 0.98 | 0.60, 1.56 | 0.918 |  |  |  |
| **Types of adjuvant therapy (Ref.**  **Other)** |  |  |  |  |  |  |
| Chemotherapy and hormonal therapy  and trastuzumab | 1.64 | 0.94, 2.85 | **0.081** |  |  |  |
| **Types of adjuvant therapy (Ref.**  **Chemotherapy or hormonal**  **therapy or trastuzumab)** |  |  |  |  |  |  |
| Other | 2.36 | 1.38, 4.05 | **0.002** | 1.82 | 0.98 – 3.38 | 0.058 |
| **Post-operative radiotherapy (Ref.**  **No)** |  |  |  |  |  |  |
| Yes | 1.29 | 0.84, 1.99 | 0.248 |  |  |  |
| NOTE. N: number of respondents included in the regression.  *Model was adjusted for participating oncology center.  ABBREVIATIONS. OR: Odds Ratio, CI: Confidence Interval, BMI: Body Mass Index, HER2: human epidermal growth factor receptor 2. | | | | | | |
